# PACT establishes a posttranscriptional brake on mitochondrial biogenesis by promoting the maturation of miR-181c

**DOI:** 10.1016/j.jbc.2022.102050

**Published:** 2022-05-19

**Authors:** Asli E. Dogan, Syed M. Hamid, Asli D. Yildirim, Zehra Yildirim, Ganes Sen, Celine E. Riera, Roberta A. Gottlieb, Ebru Erbay

**Affiliations:** 1 Smidt Heart Institute, Cedars-Sinai Medical Center, Los Angeles, California, USA; 2 Department of Molecular Biology and Genetics, National Nanotechnology Center, Bilkent University, Ankara, Turkey; 3 Department of Inflammation and Immunity, Lerner Research Institute, Cleveland Clinic, Cleveland, Ohio, USA; 4 Department of Biomedical Sciences, Center for Neural Science and Medicine, Cedars-Sinai Medical Center, Los Angeles, California, USA; 5 Department of Neurology, Board of Governors Regenerative Medicine Institute, Cedars-Sinai Medical Center, Los Angeles, California, USA; 6 David Geffen School of Medicine, University of California, Los Angeles, California, USA; 7 Department of Cardiology, Smidt Heart Institute, Cedars-Sinai Medical Center, Los Angeles, California, USA

**Keywords:** mitochondria, micro-RNA, RNAi, mitochondrial biogenesis, brown adipose tissue, ß3-AR, ß3-adrenergic receptor, BAT, brown adipose tissue, CCCP, carbonyl cyanide m-chlorophenylhydrazone, cDNA, complementary DNA, ETC, electron transfer chain, FBS, fetal bovine serum, GPx, glutathione peroxidase 1, GSS, glutathione synthetase, iWAT, inguinal WAT, MEF, mouse embryonic fibroblast, mtDNA, mitochondrial DNA, nucDNA, nuclear DNA, OCR, oxygen consumption rate, RCR, respiratory control ratio, RER, respiratory exchange ratio, RISC, RNA-induced silencing complex, ROS, reactive oxygen species, siRNA, silencer RNA, SOD, superoxide dismutase, WAT, white adipose tissue

## Abstract

The double-stranded RNA-dependent protein kinase activating protein (PACT), an RNA-binding protein that is part of the RNA-induced silencing complex, plays a key role in miR-mediated translational repression. Previous studies showed that PACT regulates the expression of various miRs, selects the miR strand to be loaded onto RNA-induced silencing complex, and determines proper miR length. Apart from PACT’s role in mediating the antiviral response in immune cells, what PACT does in other cell types is unknown. Strikingly, it has also been shown that cold exposure leads to marked downregulation of PACT protein in mouse brown adipose tissue (BAT), where mitochondrial biogenesis and metabolism play a central role. Here, we show that PACT establishes a posttranscriptional brake on mitochondrial biogenesis (mitobiogenesis) by promoting the maturation of miR-181c, a key suppressor of mitobiogenesis that has been shown to target mitochondrial complex IV subunit I (*Mtco1*) and sirtuin 1 (*Sirt1*). Consistently, we found that a partial reduction in PACT expression is sufficient to enhance mitobiogenesis in brown adipocytes in culture as well as during BAT activation in mice. In conclusion, we demonstrate an unexpected role for PACT in the regulation of mitochondrial biogenesis and energetics in cells and BAT.

The RNA-induced silencing complex (RISC) is a ribonucleoprotein complex that functions in gene silencing and miR maturation (1). RISC uses single-stranded miRs as a template to recognize complementary mRNA sequences and targets them for silencing *via* transcriptional or translational mechanisms (2, 3). The RISC consists of several RNA-binding proteins (RNAbps) including ribonuclease III (Dicer), Argonaute RISC catalytic component 2 (Ago2), transactivation response element RNA-binding protein (TRBP), and double-stranded RNA-dependent protein kinase activating protein (PRKRA or PACT) (2, 3, 4). It has been suggested that PACT is involved in efficient Dicer functioning, maturation of miRs and miR loading to the RISC (4, 5, 6, 7, 8, 9, 10, 11). Both TRBP and PACT have been proposed to determine the proper length of a subset of miRNAs (such as miR-181c that has been implicated in mitobiogenesis regulation) as well as which strand in a miRNA duplex is loaded onto the RISC (such as miR-674) (12, 13). PACT–RNA-dependent protein kinase signaling has been extensively characterized for its role in the innate immune response to viruses. Although PACT is ubiquitously expressed and is part of the RISC complex, PACT’s other function(s), especially in nonimmune cells, remain unknown (14, 15, 16, 17, 18).

A striking downregulation of PACT protein expression has been reported in mouse brown adipose tissue (BAT) upon cold-induced activation, but whether this is consequential is not known (19, 20, 21, 22). BAT activation is accompanied by dramatic gene expression changes governing mitochondrial bioenergetics and mitochondrial biogenesis (mitobiogenesis) (19, 23, 24, 25). Maintaining mitochondrial homeostasis is an interplay between clearing old/damaged mitochondria (mitophagy) and producing new/functional mitochondria (26, 27). Generation of new mitochondria is also important for maintaining healthy mitochondrial capacity that is proportional to metabolic demand. Mitobiogenesis can be triggered by numerous external stimuli such as by exercise or caloric restriction in muscle and cold in BAT (28, 29, 30). Furthermore, enhanced mitobiogenesis has been reported in human hearts upon ischemia-reperfusion injury (31). Mitobiogenesis is a complex process that requires coordination of mitochondrial DNA (mtDNA) replication, new mitochondrial protein synthesis, and protein import into the mitochondria (32, 33). Several molecular regulators of mitobiogenesis have been characterized in mammals such as transcription factors and coactivators that are responsible for coordinating mitochondrial and nuclear gene expression. Peroxisome-proliferator-activated receptor coactivator-1 α (PGC1α) is a master regulator of mitobiogenesis. Nutritional and metabolic cues are relayed to PGC1α by the NAD^+^-dependent deacetylase, SIRT1 that deacetylates and activates PGC1α. PGC1α coactivates the transcription factor, nuclear respiratory factor 1 (NRF1), which induces the mitochondrial Transcription factor A (TFAM). TFAM is required for the transcription of mtDNA (34, 35, 36). PGC1α also increases the transcription of numerous genes encoding mitochondrial proteins by coactivating peroxisome proliferator activated receptor gamma (PPARγ) (36). The PPARγ-induced Uncoupling protein 1 (UCP1) is an inner membrane protein transporter that regulates thermogenesis in BAT (37, 38). Cold or pharmacological stimulation of β_3_-adrenergic receptors (β_3_-AR) in BAT induces the cAMP pathway, which in turn activates PGC1α and downstream UCP1 production. This activation leads to a pronounced upregulation of mitobiogenesis that is coupled to thermogenesis in BAT (39).

While other RNAbps have been shown to impact adipocyte metabolism, activation, and differentiation, PACT’s role in BAT has not been investigated before (40, 41, 42, 43). Both the white adipose tissue (WAT) and BAT are major sources of exosomal miRs in mice and humans (44). Partial Dicer deficiency in BAT disrupts cold-induced thermogenesis (45). Several miRs have been identified to play a role in either activation or inhibition of BAT and subsequent thermogenesis (46, 47, 48, 49, 50, 51). Therefore, it is plausible that the observed reduction in PACT expression during BAT activation is functionally related to miR-regulated gene expression changes that occur during BAT activation (19, 20, 21, 22).

In this study, we hypothesized that the RISC component, PACT plays a key role in mitobiogenesis regulation. Our findings show that PACT establishes a brake on mitobiogenesis, in part, by modulating miR-181c expression and downregulating the expression of miR-181c′s targets that mediate transcriptional control of mitobiogenesis. PACT-deficiency leads to enhanced mitobiogenesis during BAT activation in mice. Our findings demonstrate PACT-miR-181c signaling axis is a key regulator of mitochondrial biogenesis and energetics.

## Results

### PACT is a suppressor of mitobiogenesis and energetics

PACT expression is significantly reduced upon cold-induced BAT activation (22, 40). Several RNAbps are known to impact brown adipocyte metabolism, activation, and differentiation, however, PACT’s role in BAT has not been investigated before (40, 41, 42, 43). BAT activation is accompanied by dramatic gene expression changes governing mitochondrial bioenergetics and mitochondrial biogenesis (mitobiogenesis) (19, 23, 24, 25). Therefore, we wondered whether PACT plays a role in mitochondrial biology.

**Figure 1:**
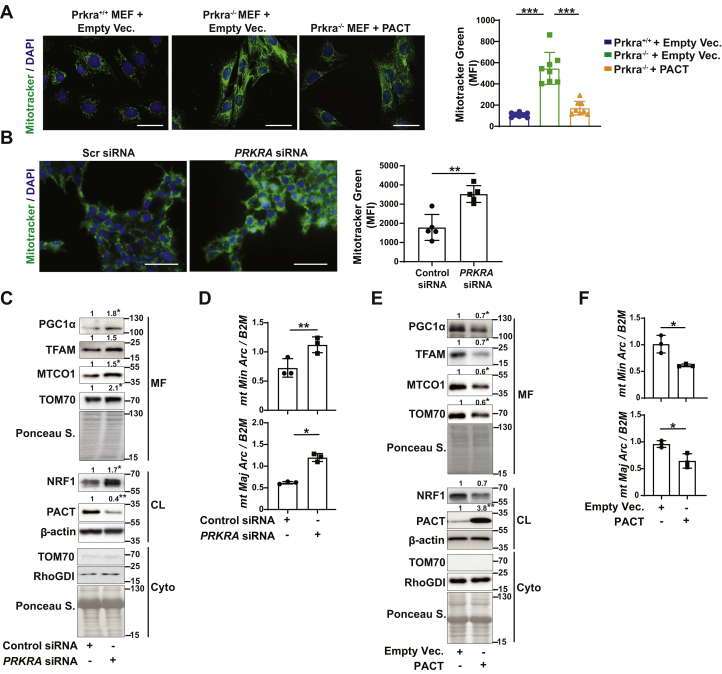
**PACT suppresses mitobiogenesis.***A* and *B*, mitochondrial mass was determined from MitoTracker Green–stained cells (quantified by measuring mean fluorescence intensity (MFI) with ImageJ from at least 200 cells; a representative image shown. The scale bar represents 50 μm): (*A*) Prkra^+/+^, Prkra^−/−^, or Prkra^−/−^ MEFs reconstituted with empty vector (Empty Vec.) or PACT (n = 8) and (*B*) HEK293T cells transfected with 100 nM scrambled or *PRKRA* siRNA (n = 5). *C* and *D*, HEK293T cells transfected with 100 nM scrambled or *PRKRA* siRNA: (*C*) Protein lysates were analyzed by Western blotting using specific antibodies for PGC1α, TOM70, antibody cocktail against ETC proteins, NRF1, PACT, and β-actin (n = 3) and (*D*) Total genomic DNA was isolated and mtDNA (*mitochondrial minor* and *major arc*): nucDNA (*B2M*) ratio was analyzed by qRT-PCR (n = 3). *E* and *F*, HEK293T cells transfected with empty vector (Empty Vec.) or PACT plasmid: (*E*) Protein lysates were analyzed by Western blotting using specific antibodies for PGC1α, TOM70, antibody cocktail against ETC proteins, NRF1, PACT, and β-actin (n = 3) and (*F*) Total genomic DNA was isolated and mtDNA (*mitochondrial minor* and *major arc*): nucDNA (*B2M*) ratio was analyzed by qRT-PCR (n = 3). Protein expression was calculated relative to β-actin (for CL) or Ponceau S. (for MF) and depicted at the top of each blot. Data are mean ± SD. Unpaired *t* test with Welch’s correction or one-way ANOVA. ∗*p* ≤ 0.05, ∗∗*p* ≤ 0.01, ∗∗∗*p* ≤ 0.001. CL, cell lysate; Cyto, cytoplasmic fraction; ETC, electron transfer chain; MEF, mouse embryonic fibroblast; MF, mitochondrial fraction; mtDNA, mitochondrial DNA; nucDNA, nuclear DNA; siRNA, silencer RNA.

To identify PACT’s role in mitochondrial biology, we began investigating whether PACT alters mitochondrial mass in cells. We observed a striking increase in mitochondrial mass (as assessed with MitoTracker Green stain) in PACT-deficient (Prkra^−/−^) mouse embryonic fibroblasts (MEFs) when compared to WT (Prkra^+/+^) MEFs. Reconstitution with PACT reduced mitochondrial mass in Prkra^−/−^ MEF, showing that mitochondrial mass is regulated by PACT (Fig. 1*A*). Additionally, mitochondrial mass was induced upon silencer RNA (siRNA)-mediated PACT knockdown in human cells (human embryonic kidney cells HEK293T) (Fig. 1*B*), demonstrating PACT’s role in regulating mitochondrial mass is conserved across species.

Since mitochondrial mass is altered as a result of PACT expression, we asked whether PACT regulates the generation of new mitochondria through mitobiogenesis. To determine the consequence of transient PACT loss-of-function on the mitobiogenesis program, we used PACT-specific siRNA to knockdown of PACT in human cells. PACT knockdown resulted in increased PGC1α, NRF1, TFAM, MTCO1, and TOM70 protein expression in human cells (Figs. 1*C* and S1*A*). This was accompanied by increased mtDNA (as assessed by mitochondrial major and minor arc) to nuclear DNA (nucDNA; as assessed by nuclear *Beta-2-Microglobulin* (*B2M*)) ratio (Fig. 1*D*). PACT overexpression, on the other hand, reduced the same mitochondrial proteins and mtDNA copy number in human cells (Figs. 1, *E* and *F* and S1*B*).

**Figure 2:**
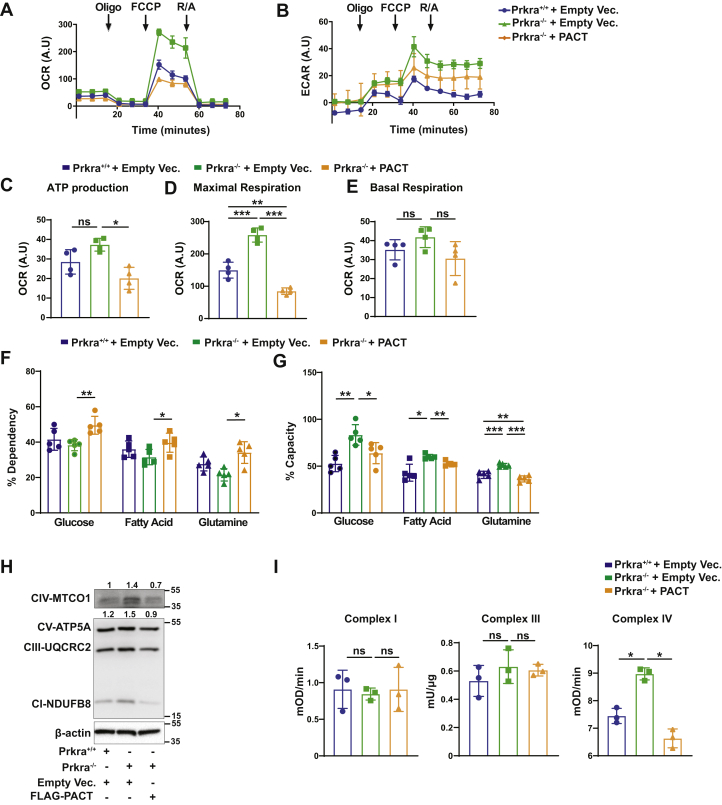
**PACT regulates mitochondrial respiratory metabolism.***A*–*E*, mitochondrial respiration was analyzed in Prkra^+/+^ or Prkra^−/−^ MEFs reconstituted with either empty vector (Empty Vec.) or FLAG-PACT by measuring (*A*) oxygen consumption rate (OCR), (*B*) extracellular acidification rate ECAR, and (*C*) ATP production after oligomycin (Oligo; 1 μM) injection. Oligomycin: ATP synthase inhibitor; FCCP: mitochondrial uncoupler; R/A: rotenone and antimycin A mix (inhibitors for ETC complex I and III, respectively). *D*, maximal respiration (as the highest OCR after FCCP injection; 1 μM). *E*, basal respiration (as OCR before oligomycin injection). *Arrows* indicate time for drug injections (n = 4; data were normalized to mitochondrial mass quantified from total OXPHOS protein levels from same samples and represented as arbitrary units (A.U)). *F* and *G*, mitochondrial fuel oxidation measurements of Prkra^+/+^, Prkra^−/−^, and Prkra^−/−^ MEFs reconstituted with FLAG-PACT to determine (*F*) dependency on and (*G*) capacity to oxidize glucose, glutamine, or fatty acids during mitochondrial respiration (n = 5). *H*, Prkra^+/+^ MEFs or Prkra^−/−^ MEFs reconstituted with either empty vector (Empty Vec.) or FLAG-PACT were analyzed by Western blotting using antibody cocktail against ETC proteins (n = 3). Protein expression was calculated relative to β-actin and depicted at the top of each blot. *I*, Prkra^+/+^ MEFs or Prkra^−/−^ MEFs reconstituted with either empty vector (Empty Vec.) or FLAG-PACT and complex I (mOD/min), III (Units/μg), and IV (mOD/min) activity was measured by ELISA (n = 3). Data are mean ± SD. Unpaired *t* test with Welch’s correction or one-way ANOVA. ∗*p* ≤ 0.05, ∗∗*p* ≤ 0.01, ∗∗∗*p* ≤ 0.001, ns: not significant. ETC, electron transfer chain; MEF, mouse embryonic fibroblast.

Next, to understand how PACT affects mitochondrial energetics, we first characterized mitochondrial oxygen consumption rates (OCRs) in Prkra^−/−^ and Prkra^+/+^ MEFs. OCR was higher in Prkra^−/−^ MEFs and this phenotype was reversed when Prkra^−/−^ MEFs were reconstituted with PACT (Fig. 2*A*). Simultaneously, extra-cellular acidification rate measurement showed high basal glycolytic activity in Prkra^−/−^ MEFs (Fig. 2*B*). Bioenergetic maps of these cells revealed that Prkra^−/−^ MEFs are highly energetic compared to Prkra^+/+^ MEFs, which was again reversed by PACT reconstitution (Fig. S1, *C* and *D*). Prkra^−/−^ MEFs displayed a tendency for increased ATP production and basal respiration but this did not reach to significancy. However, we observed a significantly higher maximal respiration that was reversed upon reconstitution of Prkra^−/−^ MEFs with PACT when OCR was normalized to mitochondrial mass (Fig. 2, *C*–*E*). When normalized to total protein levels, overall respiration (maximal and basal) and ATP production were all significantly higher in Prkra^−/−^ MEFs (Fig. S1, *E*–*I*). Furthermore, respiratory control ratio (RCR) that was calculated from OCR_FCCP_/OCR_OLIGOMYCIN_ was significantly higher in Prkra^−/−^ MEFs than in Prkra^+/+^ MEFs and reversed by PACT reconstitution (Fig. S1*J*). These results support that PACT knockout results in more efficient respiration.

We also analyzed substrate dependency and oxidation capacity of these cells. PACT deficiency did not alter dependency on glucose, fatty acids, or glutamine. Interestingly, reconstitution of Prkra^−/−^ MEFs with PACT increased dependency of these cells for all three substrates (Fig. 2*F*). Furthermore, the oxidation capacity for all types of substrates was higher in Prkra^−/−^ MEFs than in Prkra^+/+^ MEFs, and this was reversible with PACT reconstitution (Fig. 2*G*). The higher OCR and substrate oxidation capacity of Prkra^−/−^ MEFs was accompanied by significantly higher levels of MTCO1 (Figs. 2*H* and S1*K*). Similarly, siRNA-mediated PACT knockdown in human cells induced MTCO1 expression. (Fig. S1, *L* and *M*). To understand if PACT regulates the activity of mitochondrial complexes, we assessed the activity of three of the electron transfer chain (ETC) complexes, complex I, III, and IV. We observed higher complex IV activity in Prkra^−/−^ MEFs, which was decreased with PACT overexpression. No changes were observed in the activity of complex I or III (Fig. 2*I*).

These results show that mitochondrial oxygen consumption increased in parallel to the increase in mitochondrial mass in PACT-deficient cells. Furthermore, the mitochondria in Prkra^−/−^ MEFs are efficient in utilizing all types of substrates to produce ATP and do not display preference for a particular type of fuel. A possible consequence of this efficient respiration could be less reactive oxygen species (ROS) production. Consistent with this notion, ROS production was reduced in Prkra^−/−^ MEFs when compared to Prkra^+/+^ MEFs, and this was reversed by PACT reconstitution (Fig. S1*N*). These findings support a model where PACT suppression releases a posttranscriptional block on mitobiogenesis, mostly through regulating complex IV activity, upon which cells and tissues can generate more mitochondria and expand their respiratory capacity.

### PACT suppresses mitobiogenesis through miR-181c-5p

**Figure 3:**
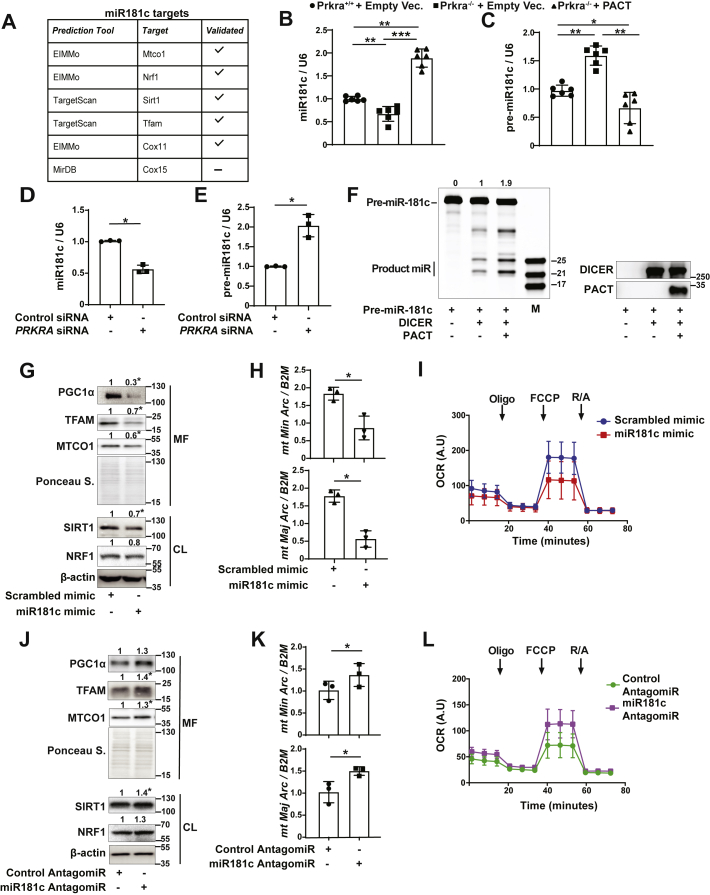

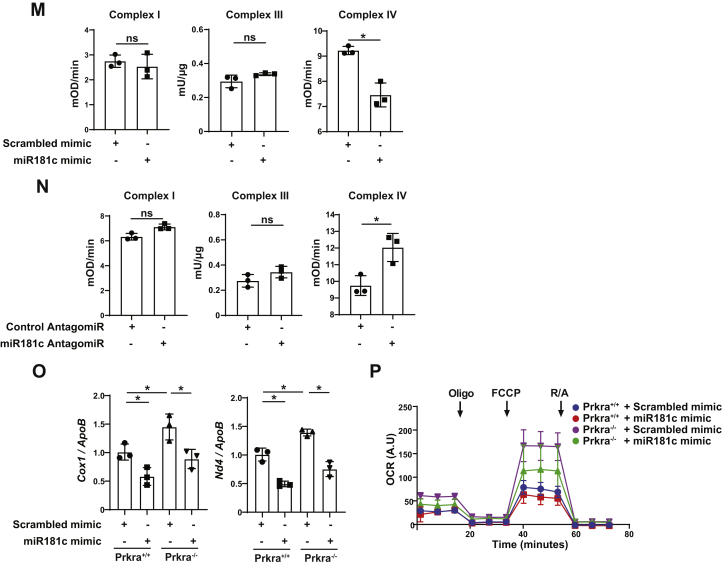
**PACT suppresses mitobiogenesis through mature miR-181c.***A*, list of mitochondrial targets of miR-181. *B* and *C*, Prkra ^+/+^ or Prkra ^−/−^ MEFs were transfected with empty vector (Empty Vec.) or FLAG-PACT and total RNA extracts were analyzed by qRT-PCR to determine (*B*) miR-181c and *U6 small nuclear RNA* (*U6*) (n = 6) and (*C*) pre-miR-181c and *U6* RNA expression (n = 6). *D* and *E*, HEK293T cells were transfected with scrambled or *PRKRA* siRNA and total RNA extracts were analyzed by qRT-PCR for (*D*) miR-181c and *U6* RNA and (*E*) pre-miR-181c and *U6* RNA expression (n = 3). *F*, *left panel*, DICER cleavage assay performed using synthetic pre-miR-181c (10 μM) as substrate with recombinant DICER or PACT (0.2 μg) at 37 °C for 4 h. Samples are separated in 15% Urea-PAGE and detected with SYBR gold staining. M indicates microRNA marker. The average band intensities for the mature miR product are indicated at the top of the gel. *Right panel*, 5 ml of same samples (in the *left panel*) were analyzed by Western blotting using specific antibodies for DICER and PACT (n = 3). *G*–*I*, HEK293T cells were transfected with scrambled or miR-181c mimic (100 nM) (n = 3); (*G*) Mitochondria enriched fraction (MF) and total cell lysates (CL) proteins were analyzed by Western blotting using specific antibodies for PGC1α, TFAM, antibody cocktail against ETC proteins, SIRT1, NRF1, and β-actin. *H*, total genomic DNA was analyzed by qRT-PCR to determine mtDNA (*mitochondrial major* and *minor arc*): nucDNA (*B2M*) ratio (n = 3). *I*, mitochondrial respiration was analyzed by OCR. *Arrows* indicate time for drug injections (n = 5; data were normalized to mitochondrial mass quantified from total OXPHOS protein levels from same samples and represented as arbitrary units (A.U)). *J*–*L*, HEK293T cells were transfected with control or miR-181c AntagomiR (100 nM). *J*, MF and CL protein lysates were analyzed by Western blotting using specific antibodies for PGC1α, TFAM, antibody cocktail against ETC proteins, SIRT1, NRF1, and β-actin (n = 3). *K*, total genomic DNA was analyzed by qRT-PCR to determine mtDNA (*mitochondrial major* and *minor arc*): nucDNA (*B2M*) ratio (n = 3). *L*, mitochondrial respiration was analyzed by OCR. *Arrows* indicate time for drug injections (n = 5; data were normalized to mitochondrial mass quantified from total OXPHOS protein levels from same samples and represented as arbitrary units (A.U)). *M*, MEF cells were transfected with scrambled or miR-181c mimic (100 nM) and complex I (mOD/min), III (Units/μg), and IV (mOD/min) activity was measured by ELISA (n = 3). *N*, MEF cells were transfected with control or miR-181c AntagomiR (100 nM) and complex I (mOD/min), III (Units/μg), and IV (mOD/min) activity was measured by ELISA (n = 3). *O* and *P*, Prkra ^+/+^ or Prkra^−/−^ MEF cells transfected with scrambled or miR-181c mimic (100 nM). *O*, total genomic DNA was analyzed by qRT-PCR for mtDNA (mitochondrial *Cox1* or *Nd4*): nucDNA (nuclear *ApoB*) ratio (n = 3). *P*, mitochondrial respiration was analyzed by OCR. *Arrows* indicate time for drug injections (n = 5; data were normalized to mitochondrial mass that was quantified from total OXPHOS protein levels from the same samples and represented as arbitrary units (A.U)). Protein expression was calculated relative to β-actin for whole cell lysate and Ponceau S. for mitochondrial fraction and depicted at the top of each blot. Data are mean ± SD. Unpaired *t* test with Welch’s correction or one-way ANOVA. ∗*p* ≤ 0.05, ∗∗*p* ≤ 0.01, ∗∗∗*p* ≤ 0.001, ns: not significant. CL, cell lysate; Cyto, cytoplasmic fraction; ETC, electron transfer chain; MEF, mouse embryonic fibroblast; MF, mitochondrial fraction; mtDNA, mitochondrial DNA; nucDNA, nuclear DNA; OCR, oxygen consumption rate; siRNA, silencer RNA.

How does the RISC-associated PACT protein regulate mitobiogenesis? Our data showed PACT regulates the expression of PGC1α, TFAM, NRF1, and MTCO1 proteins (Fig. 1). Multiple studies have shown that miR-181a and miR-181b from the miR-181 family (includes miR-181a/b/c/d that share the same seed sequence) regulate mitochondria through targeting MTCO1 and SIRT1 (13, 52, 53, 54, 55). Curiously, a previous study reported PACT plays an important role in proper length determination of miR-181c (12). Furthermore, our *in-silico* analysis of the 3′ UTR of *Tfam*, *Nrf1*, *Mtco1*, *Cox11*, and *15* as well as *Pgc1α*′s upstream regulator *Sirt1* suggested they could be regulated by miR-181c (Fig. 3*A*). Therefore, we hypothesized that PACT-regulated miR-181c could potentially execute the brake on mitobiogenesis. First, we examined the impact of PACT on mature miR-181c expression. Prkra^+/+^ MEFs had significantly lower levels of mature miR-181c than Prkra^−/−^ MEFs. Supporting this, PACT reconstitution in Prkra^−/−^ MEFs significantly induced mature miR-181c levels, but not other miR-181 family members (Figs. 3*B* and S2*A*). Simultaneously, pre-miR-181c levels were significantly higher in Prkra^−/−^ MEFs than in Prkra^+/+^ MEFs, and this was reversed by PACT reconstitution, implying PACT might regulate miR-181c′s maturation (Fig. 3*C*). As would be expected, siRNA-mediated PACT knockdown in human cells reduced miR-181c expression while increasing pre-miR-181c levels but did not affect other miR-181c family members (Figs. 3, *D* and *E* and S2, *B* and *C*).

To test the effect of PACT on pre-miR-181c processing by DICER, we performed an *in vitro* DICER cleavage assay. In this reaction, product miR-181c levels were higher when PACT was in the reaction than in DICER only condition, confirming that DICER requires PACT to optimally facilitate the maturation of pre-miR-181c (Fig. 3*F*). Altogether, these results show that PACT expression controls cellular levels of miR-181c, a miRNA implicated in mitobiogenesis regulation.

We next confirmed that miR-181c, similar to other members of the miR-181 family, can also suppress mitobiogenesis. Transfection of miR-181c mimic into human cells decreased the expression of mitobiogenesis regulators, PGC1α, TFAM, NRF1, MTCO1, and SIRT1 while simultaneously reducing mtDNA copy number (Figs. 3, *G* and *H* and S2, *D*–*F*). Additionally, miR-181c mimic significantly decreased mitochondrial OCR (normalized to mitochondrial mass or total protein levels) (Figs. 3*I* and S2, *G*–*J*). RCR was also significantly decreased in cells transfected with miR-181c mimic (Fig. S2*K*). On the other hand, inhibiting miR-181c with an antagomiR in human cells resulted in higher PGC1α, TFAM, MTCO1, NRF1, and SIRT1 protein levels with concomitant induction in mtDNA copy number (Figs. 3, *J* and *K* and S2, *L*–*N*). In parallel, miR-181c antagomiR significantly increased OCR (normalized to mitochondrial mass or total protein levels) (Figs. 3*L* and S2, *O*–*S*). RCR was also significantly increased in cells transfected with miR-181c antagomiR (Fig. S2*T*) Similar to PACT, we observed miR-181c negatively regulates complex IV activity but not complex I or III (Fig. 3, *M* and *N*). MiR-181c mimic transfection into Prkra^+/+^ MEFs and Prkra^−/−^ MEFs reduced the mtDNA copy number and mitochondrial OCR (Figs. 3, *O* and *P* and S2, *U* and *V*), which we had found to be enhanced in Prkra^−/−^ MEFs when compared to Prkra^+/+^ MEFs (Figs. 2, *A*–*E* and S1, *D* and *E*). These data support the notion that PACT exerts its effect on mitochondria through regulating of miR-181c maturation.

### Loss of PACT augments CL316,243-induced brown adipose mitobiogenesis

**Figure 4:**
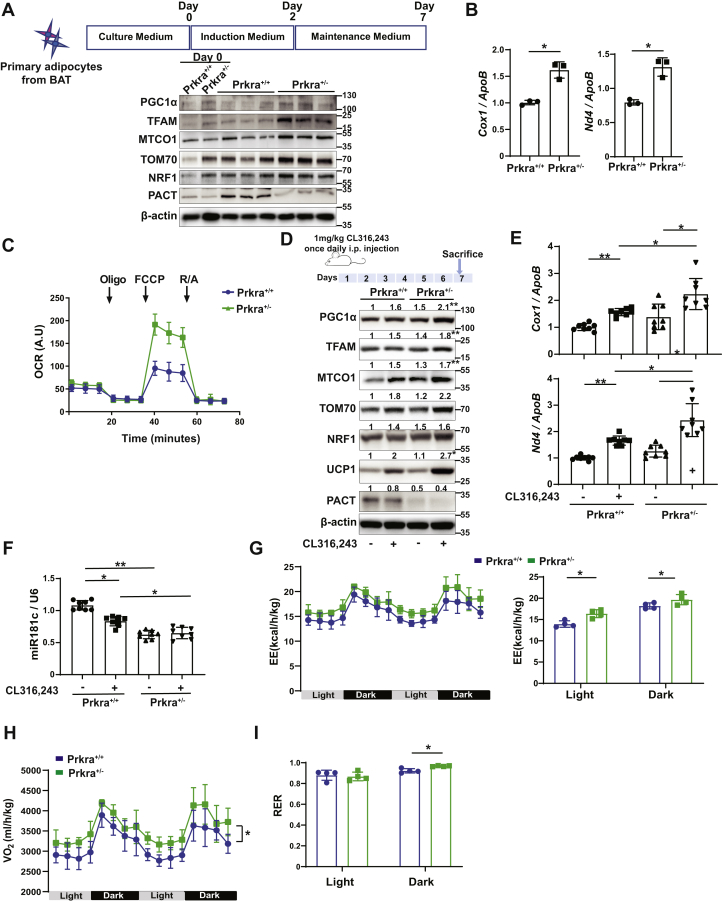
**Loss of PACT augments CL316,243-induced brown adipose mitobiogenesis.***A*, schematic representation of primary brown adipocyte differentiation protocol (*upper panel*). Mitochondrial protein levels were analyzed from protein lysates of undifferentiated (Day 0) or differentiated Prkra^+/+^ or Prkra^+/−^ brown adipocytes by Western blotting using specific antibodies for PGC1α, TFAM, antibody cocktail against ETC proteins, TOM70, NRF1, and β-actin (n = 3). *B*, the genomic DNA obtained from differentiated brown adipocytes in (*A*) was analyzed by qRT-PCR for mtDNA (*Cox1* or *Nd4*): nucDNA (*ApoB*) ratio (n = 3). *C*, mitochondrial respiration was analyzed by OCR in differentiated Prkra^+/+^ or Prkra^+/−^ brown adipocytes (n = 6; data were normalized to mitochondrial mass quantified from total OXPHOS protein levels from same samples and represented as arbitrary units (A.U)). *D*–*F*, Prkra^+/+^ or Prkra^+/−^ mice were injected with CL316,243 (1 mg/kg/day) for 6 days and sacrificed 24 h after the final injection (n = 8). *D*, BAT protein lysates were analyzed by Western blotting using specific antibodies for PGC1α, TFAM, antibody cocktail against ETC proteins, TOM70, NRF1, UCP1, and β-actin. *E*, mtDNA (*Cox1* or *Nd4*): nucDNA (*ApoB*) ratio was analyzed by qRT-PCR from total genomic DNA. *F*, total BAT RNA extracts were analyzed by qRT-PCR for miR-181c and *U6* levels. *G*–*I*, metabolic parameters of Prkra^+/+^ or Prkra^+/−^ animals (n = 4): (*G*) energy expenditure (EE), (*H*) oxygen consumption (VO_2_), and (*I*) respiratory exchange ratio (RER). Protein expression was calculated relative to β-actin (for whole cell lysate) or Ponceau S. (for mitochondrial fraction) and depicted at the top of each blot. Data are mean ± SD. Unpaired *t* test with Welch’s correction or one-way ANOVA. ∗*p* ≤ 0.05, ∗∗*p* ≤ 0.01. BAT, brown adipose tissue; ETC, electron transfer chain; mtDNA, mitochondrial DNA; nucDNA, nuclear DNA; OCR, oxygen consumption rate.

The impact of PACT expression on mitochondrial biology in animals has not been explored before. In conjunction to a published study that showed adaptive response to cold leads to reduction in PACT expression in BAT, our findings that PACT can regulate both mitobiogenesis and mitochondrial energetics prompted us to ask how PACT’s loss of function would impact mitobiogenesis during BAT activation (22, 40). While it is reported that homozygous Prkra^−/−^ mice are viable, we noted that they are smaller in size and rarely survive the neonatal period (56). In contrast, heterozygous Prkra^+/−^ mice are normal sized with a healthy appearance. Therefore, we decided to compare the mitochondria in the differentiated brown adipocytes collected from Prkra^+/−^ and Prkra^+/+^ mice. The partial loss of PACT enhanced the expression of proteins that control mitobiogenesis and increased mtDNA copy number (Figs. 4, *A* and *B* and S3, *A* and *B*).

Cold or pharmacological stimulation of β_3_-ARs in BAT induces the cAMP pathway, which in turn activates PGC1α. Working together, PGC1α and PPARγ induce the expression of UCP1 production, an inner membrane protein transporter that regulates thermogenesis in BAT (37, 38). This activation leads to a pronounced upregulation of mitobiogenesis that is coupled to thermogenesis in BAT (39). We next investigated the role of PACT in β_3_-AR–stimulated BAT activation, by using well-established pharmacologic approach to activate brown adipocytes with a β_3_-AR agonist, CL316,243 (25, 57). Brown adipocytes from Prkra^+/−^ mice respired more efficiently and induced their oxygen consumption more than brown adipocytes from Prkra^+/+^ mice (Figs. 4*C* and S3, *C*–*F*). These findings show that even a partial loss of PACT expression in brown adipocytes can lead to a marked induction in mitobiogenesis that is paralleled with increased mitochondrial respiration.

To determine PACT’s role in BAT activation *in vivo*, we stimulated BAT activation *via* β_3_-AR (1 mg/kg/day CL316,243 injection for 6 days). BAT isolated from these mice revealed that CL316,243-induced mitobiogenesis and UCP1 expression were more pronounced in Prkra^+/−^ mice in comparison to Prkra^+/+^ mice (Figs. 4*D* and S3*G*), while the histological analyses of BAT did not reveal any morphological differences between the genotypes (Fig. S3*H*). Furthermore, the mtDNA copy number was higher in Prkra^+/−^ BAT (Fig. 4*E*). Consistent with the observed PACT-dependent regulation of miR-181c expression, miR-181c levels were downregulated in Prkra^+/−^ BAT when compared to Prkra^+/+^ BAT (Fig. 4*F*). This finding is also consistent with the reduced miR-181c expression seen in PACT-deficient MEFs and upon PACT knockdown in human cells (Fig. 3).

To assess the metabolism of these mice, we performed indirect calorimetry test on Prkra^+/−^ and Prkra^+/+^ mice placed in metabolic cages. We measured energy expenditure, oxygen consumption, and respiratory exchange ratio (RER) for a duration of 48 h. Prkra^+/−^ mice showed significantly higher energy expenditure during both light and dark cycles of the day. This was also reflected in the increased oxygen consumption and RER (Fig. 4, *G*–*I*), Together, these data show that mitobiogenesis upregulation due to the partial loss of PACT protein is paired with higher metabolic rates in these animals. Furthermore, RER was significantly higher in Prkra^+/−^ mice during the dark cycle, which indicates Prkra^+/−^ mice rely more on carbohydrate metabolism than fat utilization (Fig. 4*I*). Importantly, these mice did not differ in their food and water intake, physical activity, or their lean and fat mass (Fig. S3, *I*–*K*). These collective results show that PACT plays a critical role in β_3_-AR–stimulated BAT activation and subsequent increase in energy expenditure *in vivo*.

We also explored PACT’s impact on mitochondria in inguinal WAT (iWAT) as this tissue has the capacity of expanding its mitochondria (known as beige-ing) under cold or β_3_-AR stimulation (25). In both iWAT-derived, differentiated adipocytes in culture and iWAT obtained from the CL316,243-injected mice, we observed no significant differences in key proteins for mitobiogenesis regulation (Fig. S3, *L*–*O*). While multiple other RNAbps such as HUR, Y-box binding protein 2 (Ybx2), quaking (QKI) have been shown to have an inhibitor effect on adipogenesis of both BAT and WAT (40, 58, 59), our findings show that PACT’s regulatory role in β_3_-AR–stimulated mitobiogenesis is specific to BAT.

## Discussion

Mitobiogenesis is a complex process that requires coordination of mtDNA replication, new mitochondrial protein synthesis, and protein import into the mitochondria (32, 33). Our findings reveal an unprecedented role for PACT in regulating mitobiogenesis through modulating the levels of mature miR-181c. Although being a component of the RISC, prior studies on PACT focused on its role in the innate immune response to viral infection, apoptosis, or endoplasmic reticulum stress (14, 15, 18, 60, 61). Our findings reveal that suppression of PACT and PACT-regulated miR-181c expression removes a posttranscriptional block on mitobiogenesis (as evident by the increase in the expression of key mitobiogenesis regulators, mtDNA copy number, and mitochondrial mass). Furthermore, in PACT-deficient cells, maximal respiration is increased (even after taking into account the increased mitochondrial mass in these cells), demonstrating that PACT deficiency is related to a higher respiration capacity in cells.

Mitochondrial capacity of oxidizing different fuels is linked to a flexibility in environmental conditions like feeding/fasting, exercise, or cold exposure. This allows adaptation to external stimuli and increases the ability to handle available nutrients (62, 63). Intriguingly, the mitochondria in Prkra^−/−^ MEFs were very efficient in utilizing all available substrates to produce ATP and did not display preference for a particular type of fuel. Therefore, our data suggests that depletion of PACT might be important for conditions when there is a need to adjust to fluctuating metabolic demands.

Maintaining mitochondrial homeostasis is an interplay between clearing old/damaged mitochondria and producing new/functional mitochondria (26). Adapter-mediated mitophagy is facilitated by phosphatase and tensin homolog (PTEN)-induced putative kinase 1 (PINK1) as well as E3 ubiquitin ligase Parkinson juvenile disease protein 2 (Parkin), where it ubiquitinates the target. Damage to mitochondria or a reduction in the mitochondrial membrane potential triggers PINK1 to accumulate on the mitochondrial membrane, where it acts as a sensor of mitochondrial damage. This is followed by the recruitment of Parkin, which ubiquitinates the outer mitochondrial proteins. Ubiquitinated targets are recognized *via* autophagy adapters p62 and optineurin to enable their recognition and degradation by an increase in LC3-mediated autophagosome formation (64, 65). If PACT could regulate mitophagy, this too could contribute to a change in mitochondria numbers. However, we observed no significant changes in the recruitment of Parkin or p62 to mitochondria, in the degradation of optineurin or alteration in the levels of LC3A/B in cells that were either overexpressing or deficient for PACT protein. Moreover, PACT had no impact on mitochondrial uncoupler carbonyl cyanide m-chlorophenylhydrazone (CCCP)induced mitophagy or the autophagic flux (Fig. S4, *A*–*D*). These findings support that PACT is not involved in the removal of mitochondria but controls new mitochondria production.

Efficient respiration can be coupled to less ROS production. Indeed, ROS production was reduced in Prkra^−/−^ MEFs when compared to Prkra^+/+^ MEFs, and this could be reversed by PACT reconstitution. To understand whether PACT has an impact on the antioxidant system enzymes, we also assessed the expression of catalase and peroxiredoxin 3, which convert hydrogen peroxide into water, and of superoxide dismutase (SOD) 1 and 2, which catalyze the dismutation of superoxide to yield hydrogen peroxide. Furthermore, we quantified the mRNA levels of glutathione synthetase (GSS) that is involved in glutathione biosynthesis pathway and glutathione peroxidase 1 (GPx) that can convert hydrogen peroxide to water (66). We observed no significant changes in the protein or mRNA levels of these enzymes upon overexpression or silencing of PACT in HEK293T cells (Fig. 4, *E*–*J*). Since ROS production was significantly less despite higher respiration in PACT-deficient cells than in WT cells (Fig. S1*H*), it is likely that there is more efficient OXPHOS functioning in PACT-deficient cells.

Our data supports that PACT exerts its effect on mitochondria through regulating the expression of miR-181c, likely through RISC-associated maturation. First, mature miR-181c levels are coupled to decreased pre-miR181c levels upon reconstitution of PACT in PACT-deficient cells or when PACT is overexpressed in WT cells. Second, the cleavage of pre-miR-181c by DICER was more efficient in the presence of PACT in an *in vitro* assay. In line with our current findings, a previous study has shown that PACT can modulate substrate specificity of DICER. Specifically, the study showed that DICER’s interaction with PACT or TRBP alters its activity *via* influencing the orientation of target binding and loading (8). Additionally, another study showed that depletion of PACT from RISC reduced the accumulation of mature miRs even though DICER continued to cleave pre-miRs (6). Our data in conjunction with the published data strongly supports PACT can enhance DICER cleavage activity toward specific substrates (such as pre-miR-181c) and increase the expression of mature miR product (such as miR-181c). Third, miR-181c mimic transfection into Prkra^−/−^ MEFs reduced the mtDNA copy number and mitochondrial OCR near to Prkra^+/+^ MEF levels. Collectively, these data support the notion that PACT exerts its effect on mitochondria through regulation of miR-181c maturation.

Several miR-181 family members were previously shown to target *Sirt1* and *Nrf1* to regulate mitobiogenesis and *Mtco1* to regulate mitochondrial oxygen consumption and ROS production (13, 52, 53, 54, 55). The miR-181 family has long been implicated in mitochondrial disease, where inhibition of miR181a/b was shown to protect against mitochondria-induced neurodegeneration (13). However, our data shows that PACT specifically regulates the expression of miR-181c and not the other miR-181 family members. Our data further shows that similar to PACT, the inhibition of miR-181c expression increases mitobiogenesis, whereas its overexpression suppresses mitobiogenesis. The impact of PACT can be explained, at least in part, by PACT-regulated miR-181c expression. However, the impact of miR-181c expression is not as potent as that of PACT on mitobiogenesis, implying that other miRs or regulatory factors downstream of PACT may play into the control of mitobiogenesis.

Interestingly, PACT modulates the expression miR-181c but not other miR-181 family members (Fig. S2, *A* and *B*). In addition to the previously published work by others that shows PACT affects DICER activity, our data strongly suggests that PACT has preference as to which miRs’ maturation it regulates *via* fine-tuning DICER specificity (4, 9). To decipher PACT’s impact on miR biogenesis on a global scale and in a tissue-specific manner, future studies utilizing miR-sequencing will be useful. This approach will also help further understand how PACT regulates mitochondrial respiration as miR-181c changed all OCR parameters while PACT’s effect was more pronounced on maximal respiration. This implies that PACT controls more than one miR that can regulate various aspects of OXPHOS activity.

Of note, our data indicates that PACT and miR-181c′s impact on OXPHOS activity is mostly through complex IV regulation. As complex IV is an exception for not producing ROS during reduction of oxygen (67) and its activity was significantly increased with PACT or miR-181c deletion, our data suggests that PACT exerts its effect on mitochondrial respiration and ROS production *via* impacting complex IV activity. Further investigation of the activity of all ETC complexes in the context of PACT and miR-181c is crucial to fully understand how this axis regulates mitochondrial functioning.

Notably, our findings show that PACT-deficiency can induce mitobiogenesis *in vivo* in the BAT tissue upon β_3_-AR agonist–induced activation. Our *in vivo* results imply that the PACT-mediated brake on mitobiogenesis plays a role in a physiological situation. Identification of PACT provides a key missing link in understanding RISC-mediated posttranscriptional control of mitobiogenesis. Importantly, PACT’s role in the pathophysiology in young onset, dystonia-parkinsonism disorder (dystonia 16) patients, who bear mutations in the human PACT gene, is still unknown (68, 69). The mechanistic insight into PACT’s role in mitobiogenesis regulation could explain the PACT-mediated defects in dystonias and other diseases that impact the mitochondrial energetics while simultaneously providing a novel therapeutic target to prevent such metabolic disturbances.

In summary, our findings illuminate the mechanism underlying an unprecedented molecular brake on mitobiogenesis. Our findings strongly support that the PACT-miR-181c axis-induced brake on mitobiogenesis is conserved from mouse to human cells.

## Experimental procedures

### Reagents

L-glutamine, dulbecco’s modified eagle’s medium (DMEM), PBS, fetal bovine serum (FBS), Roswell Park Memorial Institute (RPMI)-1640 medium, mirVana miR negative control mimic (4464059), mirVana-miR-181c-5p (4464067, assay ID: MC10181) and mmu-*PRKRA* silencer (AM-16708), optimal cutting temperature compound (23-730-571), Neon transfection system (MPK10096) and Neon electroporation, and Lipofectamine 3000 transfection reagent (L3000015) system were from Thermo Scientific. Trypsin, ampicillin, protease inhibitor cocktail (P8340), phosphatase inhibitor cocktail-3 (P0044), bafilomycin A1 (19–148), and CL316,243 (C5976) were from Sigma. Hematoxylin and Eosin Stain Kit (H-3502) was purchased from Vector Laboratories. miScript inhibitor negative control (1027272), anti-hsa-miR-181c-5p miR inhibitor (MIN0000674, product no: 219300), *PRKRA* siRNA (SI00054761), and all-star negative control scrambled siRNA (1027281) were purchased from Qiagen. PEI (molecular weight 25,000) was from Polysciences (23966). Antibodies: OXPHOS Rodent Antibody Cocktail (ab110413), PGC1α (ab54481), peroxiredoxin-3 (PRDX3, ab73349), and anti-SQSTM1/p62 (ab56416) were from Abcam; SIRT1 (8469S), catalase (12980T), LC3A/B (4108), parkin (Prk8,4211), and optineurin (D2L8S, 58981) were from Cell Signaling Technology; PACT (10771-1-AP), TFAM (22586-1-AP), TOM70 (14528-1-AP), and SOD2 (24127-1-AP) were from Proteintech; SOD1 (GTX100554) was from GeneTex; NRF1 (sc-33771), β-actin (linked to horse radish peroxidase) (sc-47778), Rho GDI (sc-365190), and UCP1 (sc-6528) were from Santa Cruz Biotechnology. Anti-rabbit (5450–0011) and mouse IgG (H + L) (5220–0337) were from SeraCare. Recombinant human DICER1 protein was from Creative Biomart. Recombinant human PACT protein (NBP2-51787) was from Novus Biologicals. CCCP (0452) was from Tocris.

### Primers

Hsa/Mmu_*PRKRA*_F: 5′-CAGCGGGACCTTCAGTTTG-3′

Hsa/Mmu_*PRKRA*_R: 5′-GCACATCGGATCTTTCACATTCA-3′

Mmu_*Ucp1*_F: 5′-CACCTTCCCGCTGGACACT-3′

Mmu_*Ucp1*_R: 5′-CCCTAGGACACCTTTATACCTAATGG-3′

Mmu_*Gapdh*_F: 5′-ATTCAACGGCACAGTCAAGG-3′

Mmu_*Gapdh*_R: 5′-TGGATGCAGGGATGATGTTC-3′

Hsa_*GSS*_F: 5′-GGGAGCCTCTTGCAGGATAAA-3′

Hsa_*GSS R*: 5′-GAATGGGGCATAGCTCACCAC-3′

Hsa_*GPx*_F: 5′-CAGTCGGTGTATGCCTTCTCG-3′

Hsa_*GPx R*: 5′-GAGGGACGCCACATTCTCG-3′

Hsa_*GAPDH*_F: 5′-GGAGCGAGATCCCTCCAAAAT-3′

Hsa_*GAPDH*_R: 5′-GGCTGTTGTCATACTTCTCATGG -3′

Hsa_*mt Min Arc*_F: 5′-CTAAATAGCCCACACGTTCCC-3′

Hsa *mt Min Arc*_R: 5′-AGAGCTCCCGTGAGTGGTTA-3′

Hsa_*mt Maj Arc*_F: 5′-CTGTTCCCCAACCTTTTCCT-3′

Hsa_*mt Maj Arc*_R: 5′-CCATGATTGTGAGGGGTAGG-3′

Hsa_*B2M*_F: 5′-GCTGGGTAGCTCTAAACAATGTATTCA-3′

Hsa_*B2M*_R: 5′-CCATGTACTAACAAATGTCTAAAATGGT-3′

Mmu_*Cox1*_F: 5′-TCGCCATCATATTCGTAGGAG-3′

Mmu_*Cox1*_R: 5′-GTAGCGTCGTGGTATTCCTGA-3′

Mmu_*Nd4*_F: 5′-TTATTACCCGATGAGGGAACC-3′

Mmu_*Nd4*_R: 5′-GAGGGCAATTAGCAGTGGAAT-3′

Mmu_*ApoB*_F: 5′-CGTGGGCTCCAGCATTCTA-3′

Mmu_*ApoB*_R: 5′-TCACCAGTCATTTCTGCCTTTG-3′

### Cell culture and transfections

MEF and HEK293T (American Type Culture Collection, CRL-3216) cells were grown in DMEM supplemented with 10% FBS and 1% L-glutamine in a humidified, 5% CO_2_ incubator at 37 °C.

#### Primary BAT and iWAT culture and differentiation

The stromal vascular fraction from BAT and iWAT of 6 to 8 weeks old mice were obtained by collagenase digestion, as previously described (70). Briefly, the digested was tissue centrifuged at 700*g* for 10 min. The pellet was resuspended in culture medium (10% FBS, 1% Pen-Strep DMEM) and filtered through a 70-μm cell strainer (BD BioSciences; 352350). Cells were centrifuged again at 700*g* for 10 min and plated in the same medium. After reaching confluence (day 0), cells were placed in the differentiation induction medium (DMEM with 10% FBS, 1% Pen-Strep, 5 μg/ml insulin (Sigma-Aldrich; I-0516), 1 nM triiodo-L-thyronine (T3; Sigma-Aldrich; T-2877), 2 μg/ml dexamethasone (Sigma-Aldrich; D-1756), 0.125 μM indomethacin (Sigma-Aldrich; I-7378), 0.5 mM IBMX (Sigma-Aldrich; I-5879), and 1 μM rosiglitazone (Sigma-Aldrich; R-2408). On day 2, cells were placed in the maintenance medium (DMEM with 10% FBS, 5 μg/ml insulin, and 1 nM T3) and were used on days 6 to 7 upon complete maturation.

#### Plasmid transfection

Plasmids were transfected with PEI or Lipofectamine 3000 transfection reagent into 80% confluent cells.

#### siRNA, miRNA (mimic or inhibitor) electroporations

HEK293T or MEF cells were electroporated with *PRKRA* siRNA (100 nM) or all-star negative control scrambled siRNA (100 nM), mirVana-miR-181c-5p mimic (100 nM), scrambled miR (100 nM), miR-181c-5p antagomiR (100 nM), and control antagomiR (100 nM) using the Neon electroporator (Thermo Scientific) and manufacturer-provided electroporation conditions for different cell types, as described earlier (71).

#### Bafilomycin A1 and CCCP treatments

Cells were treated with 10 μM CCCP or 50 nM bafilomycin A1 for 16 h when indicated.

### RNA isolation and qRT-PCR

Total cellular RNA was isolated by TRIsure Reagent (Bioline; BIO-38033) and reverse transcribed by using RevertAid First strand cDNA synthesis kit (ThermoScientific; K1691) to complementary DNA (cDNA), according to manufacturer’s protocol. cDNAs were amplified using specific primers and Power-Up-SYBR green (Applied Biosystems, A25742). miScript II RT kit (Qiagen; 2128160) was used for miR conversion to cDNA. miScript primer assay for mature miR-181c-5p (MS00032382), pre-miR-181c (MP00004424), and RNU6-2 (MS00033740) were from Qiagen. MiR expression analysis was performed using miScript Quantitec SYBR green kit (Qiagen; 204143). Gene expression was quantified using the relative threshold ΔΔCt method: ΔΔCt = (primer efficiency)^ˆ^(−ΔΔCt), where ΔΔCt means ΔCt (target gene) −ΔCt (reference gene), as previously described (72).

### Protein lysates, SDS/PAGE electrophoresis, transfer, and Western blotting

Cell were lysed in phospho-lysis buffer (50 mM 4-(2-hydroxyethyl)-1-piperazineethanesulfonic acid (Hepes) pH:7, 100 mM NaCl, 10 mM EDTA, 10 mM sodium fluoride, 4 mM tetra sodium pyrophosphate, 1% TritonX-100, 1× phosphatase inhibitor mixture, 1× protease inhibitor mixture). Lysates were cleared with brief centrifugation for 10 min at 8000*g*, normalized, and boiled at 95 °C after addition of 5× SDS loading dye. Proteins were then loaded to SDS/PAGE gels and transferred to PVDF membranes. Blocking and primary/secondary antibody incubation were carried out in 5% (w/v) dry milk or bovine serum albumin (in tris-buffered saline buffer with 0.1% Tween-20 (v/v)). Membranes were developed in ECL prime reagent (Amersham; RPN2236) and images were captured with ChemiDoc Imager (BioRad). Blots shown are representative of three or more experiments.

### OCR and extracellular acidification rate measurements

MEFs were cultured in X^Fe^ 96-well cell culture microplates (Agilent; 103730-100) at a density of 1 × 10^4^ cells per well in 200 μl of appropriate growth medium and incubated for 24 h at 37 °C under 5% CO_2_ atmosphere. One day before starting the assay, XF sensor cartridges were hydrated by adding 180 μl of X^Fe^ calibrant buffer to each well in the XF utility plate, the XF sensor cartridges were placed on top of the utility plate and incubated at 37 °C incubator without CO_2_ overnight. Media in the cell culture plate was removed on the day of the assay and each well was washed once with Seahorse X^Fe^ assay medium (1% L-glutamine, 1% sodium pyruvate, 1.8 mg/ml D-glucose, pH: 7.4, in Seahorse X^Fe^ DMEM medium (Agilent; 103575-100)). One hundred eighty microliters fresh Seahorse assay medium was added onto each well and the plate was incubated at 37 °C incubator without CO_2_, while the three inhibitors were loaded to the cartridge and the X^Fe^ 96-well plate. The inhibitors in Mito Stress Test Kit (Agilent; 103015-100) used to measure OCR were ATP synthase inhibitor oligomycin (1 μM), mitochondrial uncoupler carbonyl cyanide-4 (trifluoromethoxy) phenylhydrazone (1 μM) and a mixture of complex I inhibitor rotenone and complex III inhibitor antimycin A (1 μM). These three compounds were injected consecutively and OCR values with different parameters of respiration were measured and normalized to mitochondrial mass.

### Mitochondrial substrate capacity and dependency measurements

Capacity and dependency for fatty acid oxidation, glutamine, and pyruvate were measured with Seahorse Fuel Flex Kit (Agilent; 103260-100) *via* injection of Etomoxir (2 μM), Bis-2-(5-phenylacetamido-1,3,4-thiadiazol-2-yl) ethyl sulfide (3 μM), and UK5099 (4 μM), respectively. OCR was measured using a Seahorse X^Fe^96. Capacity and dependency on these pathways were calculated by using Wave software.

### MitoTracker staining

MitoTracker Green FM (Invitrogen; M7514) was used to stain mitochondria according to the manufacturer’s protocols. Cells were fixed with 4% paraformaldehyde, mounted onto slides with Fluoroshield mounting medium with DAPI (Abcam; ab104139), visualized with Leica TCS SP5 X Confocal Microscope at 60× magnification, and mean fluorescence intensity was calculated in ImageJ software.

### mtROS measurement

Mitochondrial ROS was assessed by incubating the cells with 5 μM mitoSOX red (Invitrogen; M36008), followed by flow cytometry analysis. ROS levels were quantified as the mean fluorescence intensity using BD Fortessa (BD Biosciences) and FACSDiva software with compensation controls acquired on the same day.

### Assessment of mtDNA copy number

Cells were scraped in DNA Lysis Buffer (10 mM NaCl, 20 mM Tris pH:6, 1 mM EDTA, 10% SDS) and incubated at 37 °C for 2 h. UltraPure Phenol:Chloroform:Isoamyl Alcohol (Thermo Scientific; 15-593-031) was added to induce phase separation (at 12,000*g* for 15 min). The DNA containing transparent upper phase was collected into a new tube, mixed with chloroform, and centrifuged (at 12,000*g* for 5 min). The clear upper phase was collected to a new tube and DNA was precipitated by the addition of absolute ethanol containing sodium acetate (1/10 volume) followed by centrifugation (at 12,000*g* for 20 min). Precipitated DNA was dissolved in water. DNA (0.2 μg) was amplified using nuclear- or mitochondria-encoded genes-specific primers and Power-Up-SYBR green (Applied Biosystems; A25742). mtDNA: nucDNA ratios were calculated by normalizing results of mitochondria-encoded gene to nuclear-encoded gene.

### Mitochondrial enrichment

Cells were homogenized in mitochondrial isolation buffer (250 mM sucrose, 1 mM EDTA, 10 mM Hepes, pH 7.4) containing inhibitors (1× phosphatase inhibitor cocktail 3 and 1× protease inhibitor cocktail) by running through 27.5 g needle three times. Nuclei and unbroken cells were eliminated by low-speed spin (600*g*, 4 °C, 5 min). A small portion of the supernatant was saved (cell lysate) and the rest was centrifuged (7000*g*, 4 °C, 15 min) to obtain the final mitochondria-enriched pellet and supernatant (cytosol). The mitochondria-enriched pellet was resuspended in isolation buffer and centrifuged (7000*g*, 4 °C, 5 min) as a final wash. The pellet was resuspended in cold phospho-lysis buffer with inhibitors. Protein concentrations are measured with DC Protein Assay Kit II (Bio-Rad; 500-0112). Normalized samples are boiled in SDS loading dye at 95 °C for 5 min before loading on SDS/PAGE gels. After separation according to protein molecular weights on these gels, samples were transferred to nitrocellulose membranes. Membranes are then stained with Ponceau S solution (Sigma-Aldrich; P7170-1L), washed with distilled water, and imaged, as loading control.

### Measurements of complex I, III, and IV activities

Measurements of complex I activity (Complex I Enzyme Activity Microplate Assay Kit, ab109721, Abcam), complex III activity (Mitochondrial Complex III Activity Assay Kit, ab287844, Abcam), and complex IV activity (Complex IV Enzyme Activity Microplate Assay Kit, ab109911, Abcam) were done in MEF cells according to the manufacturer’s instructions. To measure complex I and IV activity, cells were harvested and loaded onto the plate for 3 h. Two hundred microliters assay solution was added to measure optical density in kinetic mode for the indicated times. Activity is indicated as the change in absorbance per minute (mOD/min). Complex III activity is calculated as Δ*C* Δ*t* × *p* × D (Units/μg) where ΔC is the change in reduced cytochrome c concentration during Δt, Δt is the time duration between t1 and t2 (min), p is the mitochondrial protein sample (μg), and D is the dilution factor.

### *In vitro* DICER cleavage assay

Pre-miR-181c (5′-AACAUUCAACCUGUCGGUGAGUUUGGGCAGCUCAGGCAAACCAUCGACCGUUGAGUGGACC-3′) was custom synthesized by IDT DNA technologies. DICER cleavage reaction was performed in cleavage assay buffer (20 mM Tris–HCl (pH 6.5), 1.5 mM MgCl_2_, 25 mM NaCl, 1 mM DTT, and 1% glycerol) with DICER (0.2 μg) or PACT (0.2 μg) and synthetic pre-miR-181c (10 μM) at 37 °C for 4 h. Reaction was stopped by adding an equal volume of RNA loading dye (NEB) and heating at 70 °C for 5 min followed by resolving on 15% urea-acrylamide gel. Gel was stained with SYBR Gold and imaged using ChemiDoc imager (BioRad).

### Animals

All procedures were approved by the Institutional Animal Care and Use Committee of Cedars-Sinai Medical Center. Mice were housed at 22 °C with a 12 h light/12 h dark cycle and fed with regular Chow diet. Sperm of Prkra^−/−^ mice was purchased from Jackson Laboratories and mice were redrived at the Cedars-Sinai Medical Center Animal Models Core Facility. Age matched C57BL/6J littermates were used as controls.

### CL316,243 treatment

For *in vivo* experiments, CL316,243 (1 mg/kg body weight) or vehicle control (sterile saline) was injected intraperitoneally once a day for 6 days. Mice were sacrificed 24 h after the last injection. Adipose tissue depots, iWAT, and BAT were harvested for protein, DNA, RNA, and histological analyses.

### Metabolic cages

Mice were single housed in the Phenomaster System (TSE Systems) for a total of 3 days with a 24-h acclimation period. During the 48 h of data collection, airflow, temperature, oxygen and carbon dioxide content, oxygen uptake, carbon dioxide production, food, water intake, and locomotor activity were measured simultaneously. Energy expenditure and RER were calculated automatically from the oxygen uptake and carbon dioxide production. Data were collected with the instrument software and exported to Excel.

### EchoMRI

Whole body composition of mice was detected *via* the EchoMRI system (EchoMRI, LLC), for fat and lean mass. Animal was placed in a clear plastic holder without anesthesia. The holder was then inserted into the tubular space of the EchoMRI system for the animal to be scanned.

### Histological analysis

BATs were fixed for 24 h at room temperature in PBS containing 4% paraformaldehyde. Following sucrose gradient to cryopreserve the tissues, they were embedded in optimal cutting temperature compound. Tissue sections (8 μm) were used for H&E staining.

### Statistics

GraphPad Prism 8 (GraphPad Software, Inc) was used to statistically analyze data and create graphs. Results are reported as mean ± SD. Statistical significance was determined with Student’s *t* test with Welch’s correction for comparison of two groups or one-way ANOVA for comparison of multiple groups. *p* < 0.05 was considered as ∗ significant.

## Data availability

All data are available in the main text or the supplementary materials. Research materials used in the article can be requested from authors.

## Supporting information

This article contains supporting information.

## Conflict of interest

The authors declare that they have no competing interests.

## Supporting information

Supplemental Figure S1A–J **PACT regulates mitobiogenesis and mitochondrial oxygen consumption.***A*, quantifications of band intensities for PGC1α, TOM70, MTCO1 and NRF1 from blots in Figure 1*C*. *B*, quantifications of band intensities for PGC1α, TFAM, OXPHOS, TOM70 and NRF1 relative to Ponceau S (for MF) and β-actin (for CL) from blots in Figure 1*E*. *C*, energy map for Prkra^+/+^, Prkra^−/−^ MEFs and Prkra^−/−^ MEFs reconstituted with FLAG-PACT calculated from OCR. Cells were categorized according to their bioenergetics, where high OCR and ECAR corresponds to an energetic state and low OCR and ECAR indicates quiescence. *D*, protein lysates from cells in Figure 2 were analyzed by Western blotting using specific antibodies for PACT and β-actin to confirm transfection efficiency. *E*–*I*, mitochondrial respiration was analyzed in Prkra^+/+^ or Prkra^−/−^ MEFs reconstituted with either empty vector (Empty Vec.) or FLAG-PACT by measuring (*E*) oxygen consumption rate (OCR) (*F*) extracellular acidification rate ECAR, and (*G*) ATP production after oligomycin (Oligo; 1 μM) injection. Oligomycin: ATP synthase inhibitor; FCCP: mitochondrial uncoupler; R/A: rotenone and antimycin A mix (inhibitors for ETC complex I and III, respectively). *H*, maximal respiration (as the highest OCR after FCCP injection; 1 μM). *I*, basal Respiration (as OCR before oligomycin injection). *Arrows* indicate time for drug injections (n = 5; data were normalized to μg of total protein). *J*, respiratory control ratio (RCR) of Prkra^+/+^ or Prkra^−/−^ MEFs reconstituted with either empty vector (Empty Vec.) or FLAG-PACT was calculated from the ratio of State 3 to State 4 (OCR_FCCP_ to OCR_OLIGOMYCIN_) (n = 4). *K*, quantification of band intensities for MTCO1 and total ETC complex relative β-actin in blots from Figure 2*H*. *L*, scrambled or *PRKRA* siRNA were transfected into HEK293T cells. Protein lysates were analyzed by Western blotting using antibodies specific for PACT, ETC and β-actin (n = 3). *M*, quantification of band intensities for MTCO1 and total ETC relative to β-actin in blots from Fig. S2*G*. *N*, flow cytometry measurements for mitochondrial ROS stained with mitoSOX Red mitochondrial superoxide indicator from Prkra^+/+^, Prkra^−/−^ MEFs and Prkra^−/−^ MEFs reconstituted with FLAG-PACT. Data are mean ± SD. Unpaired *t*-test with Welch’s correction or one-way ANOVA. ∗*p* ≤ 0.05, ∗∗*p* ≤ 0.01, ∗∗∗*p* ≤ 0.001.

Supplemental Figure S1K–N

Supplemental Figure S2A–J **PACT’s control of mitobiogenesis is through miR-181c.***A*, total RNA from Prkra^+/+^ or Prkra^−/−^ MEFs that were transfected with FLAG-PACT plasmid or Empty Vec. was analyzed by qRT-PCR for miR-181a, b, d and *U6* by qRT-PCR (n = 6). *B*, total RNA from HEK293 cells that were transfected with scrambled or *PRKRA* siRNA (100 nM) was analyzed by qRT-PCR for miR-181a, b, d and *U6* (n = 5). *C*, *PRKRA* and *GAPDH* mRNA were analyzed by qRT-PCR from total RNA in Figure 3, *D* and *E*. *D*, related to Figure 3*G* (n = 3): Protein lysates were analyzed by Western blotting using specific antibodies for TOM70 and RhoGDI. *E* and *F*, related to Figure 3, *G*–*I*: Total RNA was analyzed by qRT-PCR for (*E*) miR-181c and *U6* RNA, (*F*) Quantifications of band intensities for PGC1α, TFAM, MTCO1, NRF1 and SIRT1 relative to Ponceau S (for MF) and β-actin (for CL) (*G*) Related to Figure 3*I*: Total RNA was analyzed by qRT-PCR for miR-181c and *U6* (n = 3). *H*, related to Figure 3*I*: ATP production after oligomycin (Oligo; 1 μM) injection, Maximal respiration (as the highest OCR after FCCP injection; 1 μM) and Basal Respiration (as OCR before oligomycin injection) calculated from MEFs transfected with scrambled or miR-181c-5p mimic (100 nM) (n = 5; data were normalized to mitochondrial mass quantified from total OXPHOS protein levels (right panel) from same samples and represented as arbitrary units (A.U)). *I* and *J*, HEK293T cells were transfected with scrambled or miR-181c mimic (100 nM); (*I*) OCR was measured (n = 5; data were normalized to μg of total protein). *J*, ATP production after oligomycin (Oligo; 1 μM) injection, Maximal respiration (as the highest OCR after FCCP injection; 1 μM) and Basal Respiration (as OCR before oligomycin injection) were calculated. *K*, respiratory control ratio (RCR) of HEK293T cells transfected with scrambled or miR-181c mimic (100 nM) was calculated from the ratio of State 3 to State 4 (OCR_FCCP_ to OCR_OLIGOMYCIN_) (n = 5). *L*, related to Figure 3*J*: Protein lysates were analyzed by Western blotting using specific antibodies for TOM70 and RhoGDI (n = 3). *M*–*O*, related to Figure 3*J*: Total RNA was analyzed by qRT-PCR for (*M*) miR-181c-5p and *U6* RNA (n = 3), (*N*) Quantifications of band intensities for PGC1α, TFAM, MTCO1, NRF1 and SIRT1 relative to Ponceau S. (for MF) and β-actin (for CL) in blots from Figure 3*J*. *O*, related to Figure 3*L*: Total RNA was analyzed by qRT-PCR for miR-181c and U6 (n = 3). *P*, related to Figure 3*L*: ATP production after oligomycin (Oligo; 1 μM) injection, Maximal respiration (as the highest OCR after FCCP injection; 1 μM) and Basal Respiration (as OCR before oligomycin injection) calculated from Prkra^+/+^ MEFs transfected with control or miR-181c-5p antagomiR (100 nM) (n = 5; data were normalized to mitochondrial mass quantified from total OXPHOS protein levels (*right panel*) from same samples and represented as arbitrary units (A.U)). *R*–*T*, HEK293T cells were transfected with control or miR-181c antagomiR (100 nM); (*R*) OCR was measured (n = 5; data were normalized to μg of total protein). *S*, ATP production after oligomycin (Oligo; 1 μM) injection, Maximal respiration (as the highest OCR after FCCP injection; 1 μM) and Basal Respiration (as OCR before oligomycin injection) were calculated. *T*, respiratory control ratio (RCR) of HEK293T cells transfected with control or miR-181c antagomiR (100 nM) was calculated from the ratio of State 3 to State 4 (OCR_FCCP_ to OCR_OLIGOMYCIN_) (n = 5). *U*, related to Figure 3, *O* and *P*: total RNA analyzed by qRT-PCR for miR181c and *U6* (n = 3). *V*, related to Figure 3*P*: ATP production after oligomycin (Oligo; 1 μM) injection, Maximal respiration (as the highest OCR after FCCP injection; 1 μM) and Basal Respiration (as OCR before oligomycin injection) calculated from Prkra^+/+^ and Prkra^−/−^ MEFs transfected with scrambled or miR-181c-5p mimic (100 nM) (n = 6; data were normalized to mitochondrial mass quantified from total OXPHOS protein levels (*right panel*) from same samples and represented as arbitrary units (A.U)). Data are mean ± SD. Unpaired t-test with Welch’s correction or one-way ANOVA. ∗*p* ≤ 0.05, ∗∗*p* ≤ 0.01, ∗∗∗*p* ≤ 0.001, ns: not significant. Cyto, Cytoplasmic fraction; CL, Cell lysate; MF, mitochondrial fraction.

Supplemental Figure S2K–S

Supplemental Figure S2T–V

Supplemental Figure S3A–G **PACT is required for BAT but not iWAT mitobiogenesis.***A*, related to Figure 4*A*: Primary mouse brown adipocyte total mRNA was analyzed by qRT-PCR for *Ucp1* and *Gapdh* mRNA expression (n = 3). *B*, related to Figure 4*A*: Quantification of band intensities for PGC1α, TFAM, MTCO1, TOM70, PACT and NRF1 relative to β-actin. *C*, related to Figure 4*C*: ATP production after oligomycin (Oligo; 1 μM) injection, Maximal respiration (as the highest OCR after FCCP injection; 1 μM) and Basal Respiration (as OCR before oligomycin injection) calculated from Prkra^+/+^ or Prkra^+/−^ brown adipocytes (n = 4; data were normalized to mitochondrial mass quantified from total OXPHOS protein levels (*right panel*) from same samples and represented as arbitrary units (A.U)). *D*–*F*, mitochondrial respiration was analyzed in Prkra^+/+^ or Prkra^+/−^ primary mouse brown adipocytes by (*D*) oxygen consumption rate (OCR) (*E*) ATP production after oligomycin (Oligo; 1 μM) injection. Oligomycin: ATP synthase inhibitor; FCCP: mitochondrial uncoupler; R/A: rotenone and antimycin A mix (inhibitors for ETC complex I and III, respectively), Maximal respiration (as the highest OCR after FCCP injection; 1 μM) and Basal Respiration (as OCR before oligomycin injection). Arrows indicate time for drug injections (n = 6; data were normalized to μg of total protein). *F*, respiratory control ratio (RCR) was calculated from the ratio of State 3 to State 4 (OCR_FCCP_ to OCR_OLIGOMYCIN_) (n = 4). *G*, quantifications of band intensities for PGC1α, TFAM, MTCO1, TOM70, NRF1, PACT and UCP1 relative to β-actin in blots from Figure 4*D* (n = 8). *H*–*K*, related to mice in Figure 4*D*: (*H*) Representative H&E stained images of BAT (n = 8) (Scale bar = 50 μm). *I*, the water and food intake, (*J*) physical activity, (*K*) lean and fat mass of mice in Figure 4 (n = 4). *L*, total cell protein lysates of undifferentiated (Day 0) or differentiated iWAT adipocytes from Prkra^+/+^ or Prkra^+/−^ mice were analyzed by Western blotting using specific antibodies for PGC1α, TFAM, OXPHOS, TOM70, NRF1, PACT and β-actin (n = 3). *M*, RNA extracts from the experiment in *I* was analyzed by qRT-PCR for *Ucp1* and *Gapdh* mRNA (n = 3). *N*, protein lysates from iWAT of Prkra^+/+^ or Prkra^+/−^ animals that were injected with saline or CL316,243 (1 mg/kg/d) were analyzed by Western blotting using antibodies specific for PGC1α, TFAM, OXPHOS, TOM70, NRF1, PACT, UCP1 and β-actin (n = 8). *O*, quantification of band intensities for relative to β-actin in blots from *K* (n = 8). Data are mean ± SD. Unpaired *t*-test with Welch’s correction or one-way ANOVA. ∗*p* ≤ 0.05, ∗∗*p* ≤ 0.01, ∗∗∗*p* ≤ 0.001.

Supplemental Figure S3H–O

Supplemental Figure S4A–D **PACT does not impact mitophagy or ROS scavenging enzyme protein levels.***A*, protein lysates from HEK293T cells that were transfected with empty vector (Empty Vec.) or FLAG-PACT plasmid and treated with CCCP (10 μM; 16 h) or Bafilomycin A1 (Baf) (50 nM; 16 h) were analyzed by Western blotting using specific antibodies for Optineurin, Parkin, p62, LC3A/B, PACT and β-actin (n = 3). *B*, quantification of the band intensities in (*A*): Optineurin, Parkin, p62 and LC3-II relative to Ponceau S. and PACT relative to β-actin (n = 3). *C*, protein lysates from HEK293T cells that were transfected with 100 nM Scrambled or *PRKRA* siRNA and treated with CCCP (10 μM; 16 h) or Bafilomycin A1 (50 nM; 16 h) were analyzed by Western blotting using specific antibodies for Optineurin, Parkin, p62, LC3A/B, PACT and β-actin (n = 3). *D*, quantification of band intensities in (*C*): Optineurin, Parkin, p62 and LC3-II relative to Ponceau S. and PACT relative to β-actin (n = 3). *E*–*G*, HEK293T cells were transfected with empty vector (Empty Vec.) or FLAG-PACT plasmid: (*E*) were analyzed by Western blotting using specific antibodies for CATALASE, SOD1, SOD2, PRDX3, PACT and β-actin (n = 4). *F*, quantifications of band intensities in (*E*) for CATALASE, SOD1, SOD2, PRDX3 and PACT were relative to β-actin. *G*, total RNA was analyzed by qRT-PCR for *Glutathione synthetase* (*GSS*), *Glutathione peroxidase* (*GPx*) and *GAPDH* mRNA expression (n = 5). *H*–*J*, HEK293T cells were transfected with 100 nM Scrambled or *PRKRA* siRNA: (*H*) Protein lysates were analyzed by Western blotting using specific antibodies for CATALASE, SOD1, SOD2, PRDX3, PACT and β-actin (n = 4). *I*, quantifications of band intensities (in *G*) for CATALASE, SOD1, SOD2, PRDX3 and PACT were relative to β-actin. *J*, total RNA was analyzed by qRT-PCR for *Glutathione synthetase* (*GSS*), *Glutathione peroxidase* (*GPx*) and *GAPDH* mRNA expression (n = 5). Data are mean ± SD. Unpaired *t*-test with Welch’s correction. ∗*p* ≤ 0.05, ∗∗*p* ≤ 0.01, ∗∗∗*p* ≤ 0.001, ns: not significant. CL, Cell lysate; Cyto, Cytoplasmic fraction; MF, Mitochondrial fraction.

Supplemental Figure S4E–J
