# Supplementary figures and images for: PACT establishes a posttranscriptional brake on mitochondrial biogenesis by promoting the maturation of miR-181c
Source: J Biol Chem. 2022 May 19;298(7):102050. doi: 10.1016/j.jbc.2022.102050 (PMC9218515; doi:10.1016/j.jbc.2022.102050)

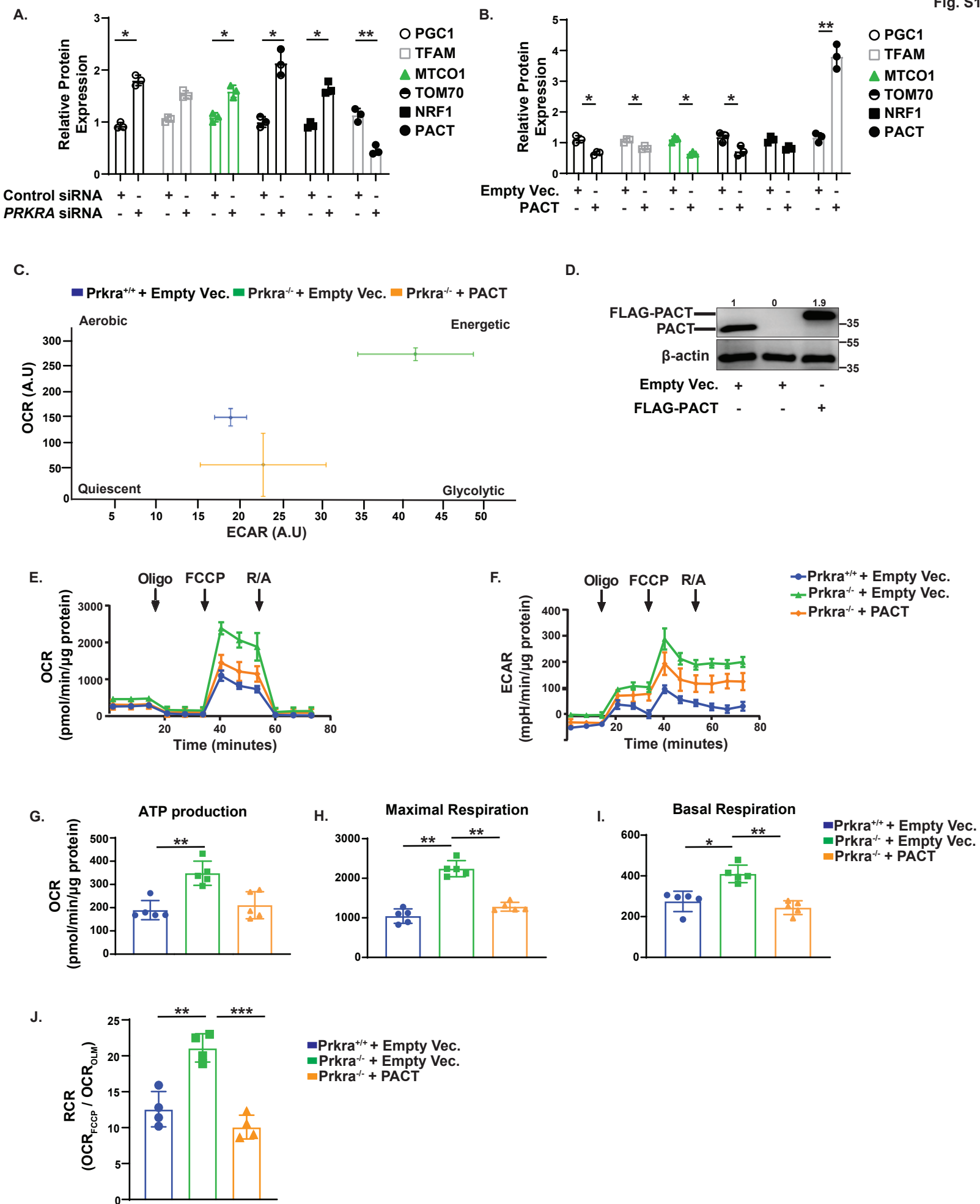

Supplement: Supplemental Figure S1A–J — PACT regulates mitobiogenesis and mitochondrial oxygen consumption.A, quantifications of band intensities for PGC1α, TOM70, MTCO1 and NRF1 from blots in Figure 1C. B, quantifications of band intensities for PGC1α, TFAM, OXPHOS, TOM70 and NRF1 relative to Ponceau S (for MF) and β-actin (for CL) from blots in Figure 1E. C, energy map for Prkra+/+, Prkra−/− MEFs and Prkra−/− MEFs reconstituted with FLAG-PACT calculated from OCR. Cells were categorized according to their bioenergetics, where high OCR and ECAR corresponds to an energetic state and low OCR and ECAR indicates quiescence. D, protein lysates from cells in Figure 2 were analyzed by Western blotting using specific antibodies for PACT and β-actin to confirm transfection efficiency. E–I, mitochondrial respiration was analyzed in Prkra+/+ or Prkra−/− MEFs reconstituted with either empty vector (Empty Vec.) or FLAG-PACT by measuring (E) oxygen consumption rate (OCR) (F) extracellular acidification rate ECAR, and (G) ATP production after oligomycin (Oligo; 1 μM) injection. Oligomycin: ATP synthase inhibitor; FCCP: mitochondrial uncoupler; R/A: rotenone and antimycin A mix (inhibitors for ETC complex I and III, respectively). H, maximal respiration (as the highest OCR after FCCP injection; 1 μM). I, basal Respiration (as OCR before oligomycin injection). Arrows indicate time for drug injections (n = 5; data were normalized to μg of total protein). J, respiratory control ratio (RCR) of Prkra+/+ or Prkra−/− MEFs reconstituted with either empty vector (Empty Vec.) or FLAG-PACT was calculated from the ratio of State 3 to State 4 (OCRFCCP to OCROLIGOMYCIN) (n = 4). K, quantification of band intensities for MTCO1 and total ETC complex relative β-actin in blots from Figure 2H. L, scrambled or PRKRA siRNA were transfected into HEK293T cells. Protein lysates were analyzed by Western blotting using antibodies specific for PACT, ETC and β-actin (n = 3). M, quantification of band intensities for MTCO1 and total ETC relative to [file mmc1.pdf]

K.

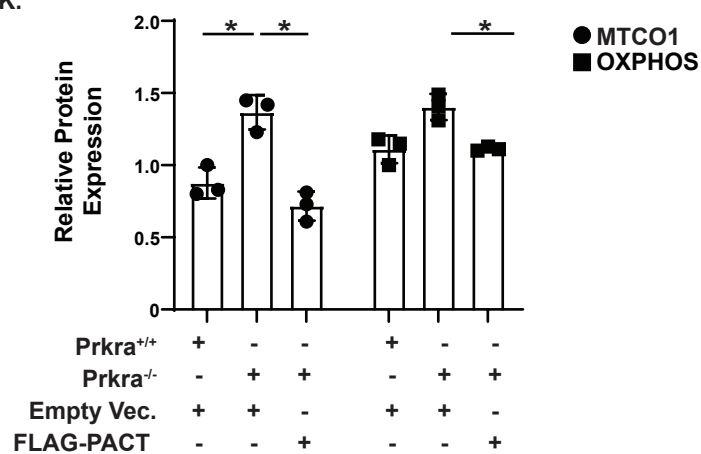

L.

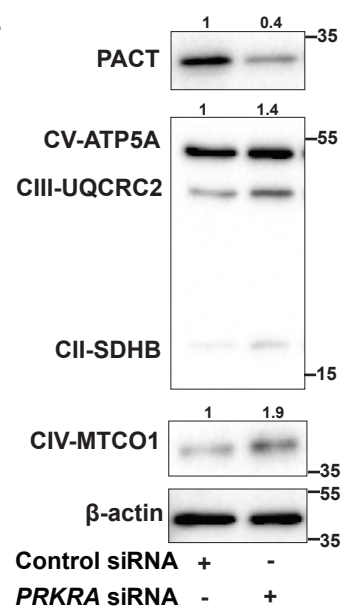

M.

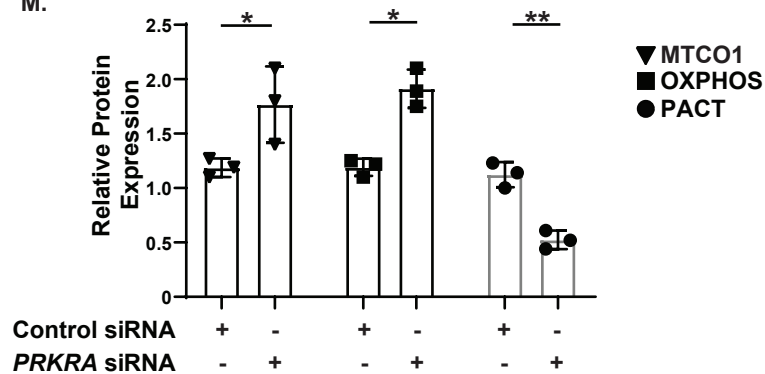

N.

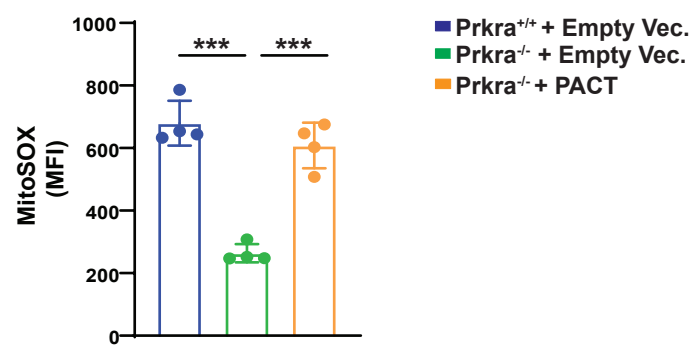

Supplement: Supplemental Figure S1K–N [file mmc2.pdf]

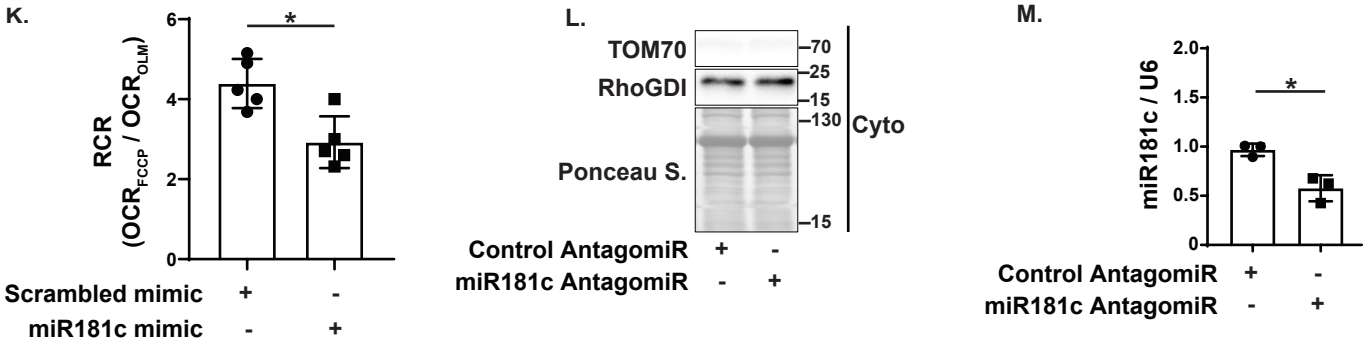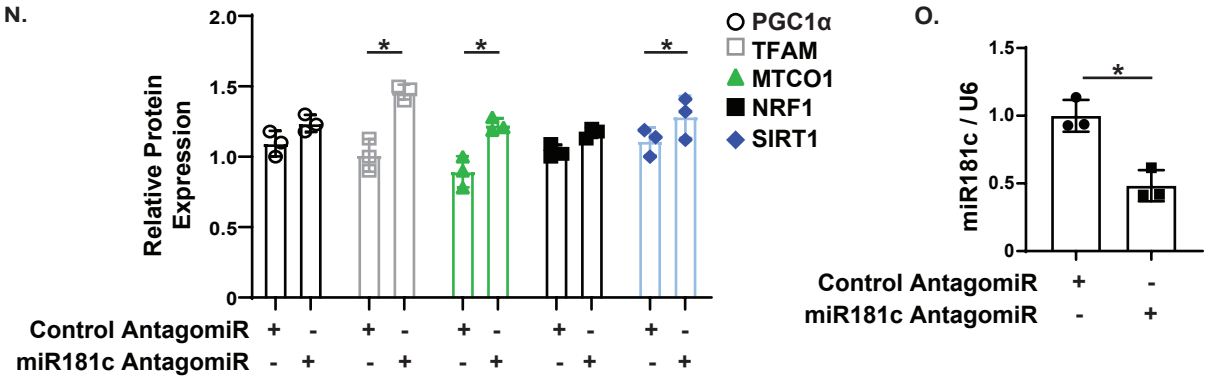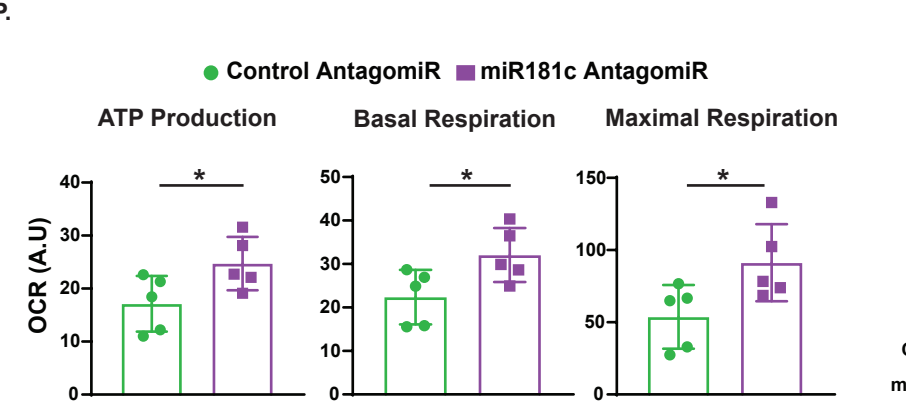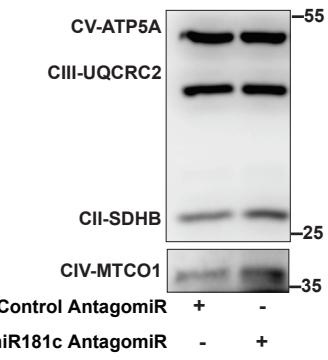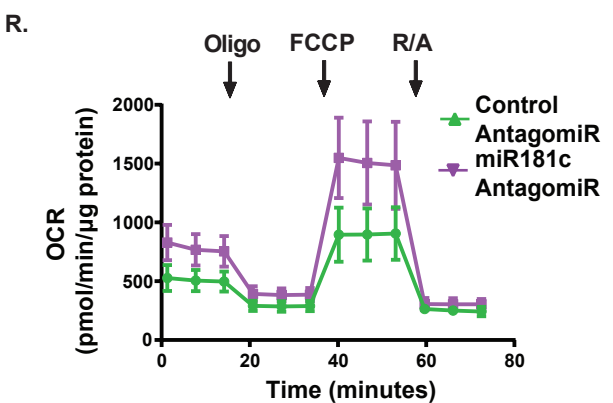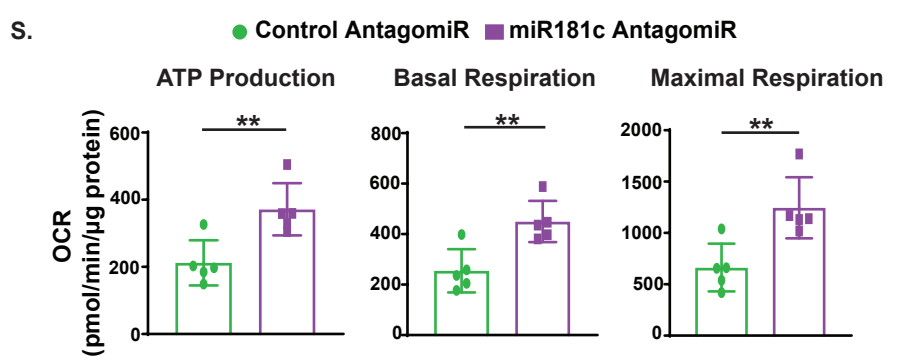

Supplement: Supplemental Figure S2K–S [file mmc4.pdf]

T.

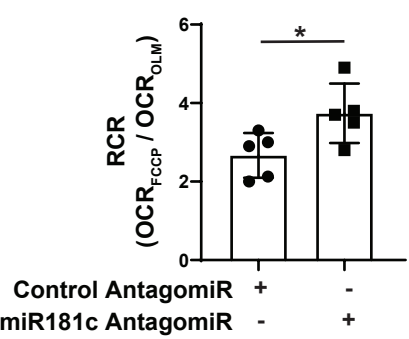

U.

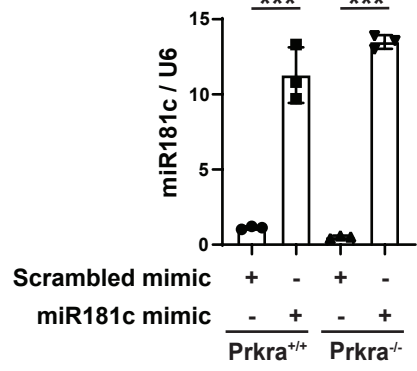

V.

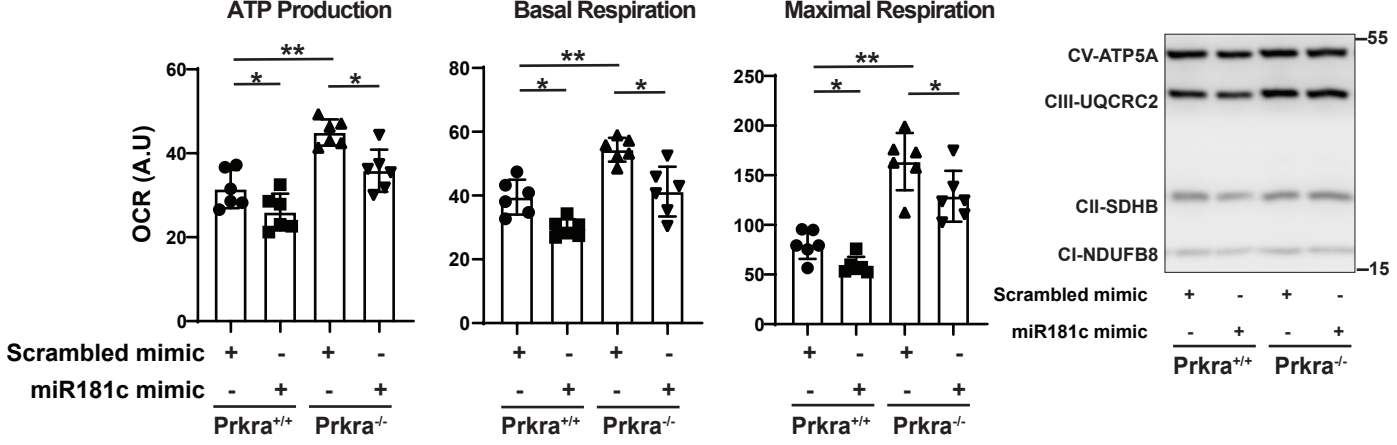

Supplement: Supplemental Figure S2T–V [file mmc5.pdf]

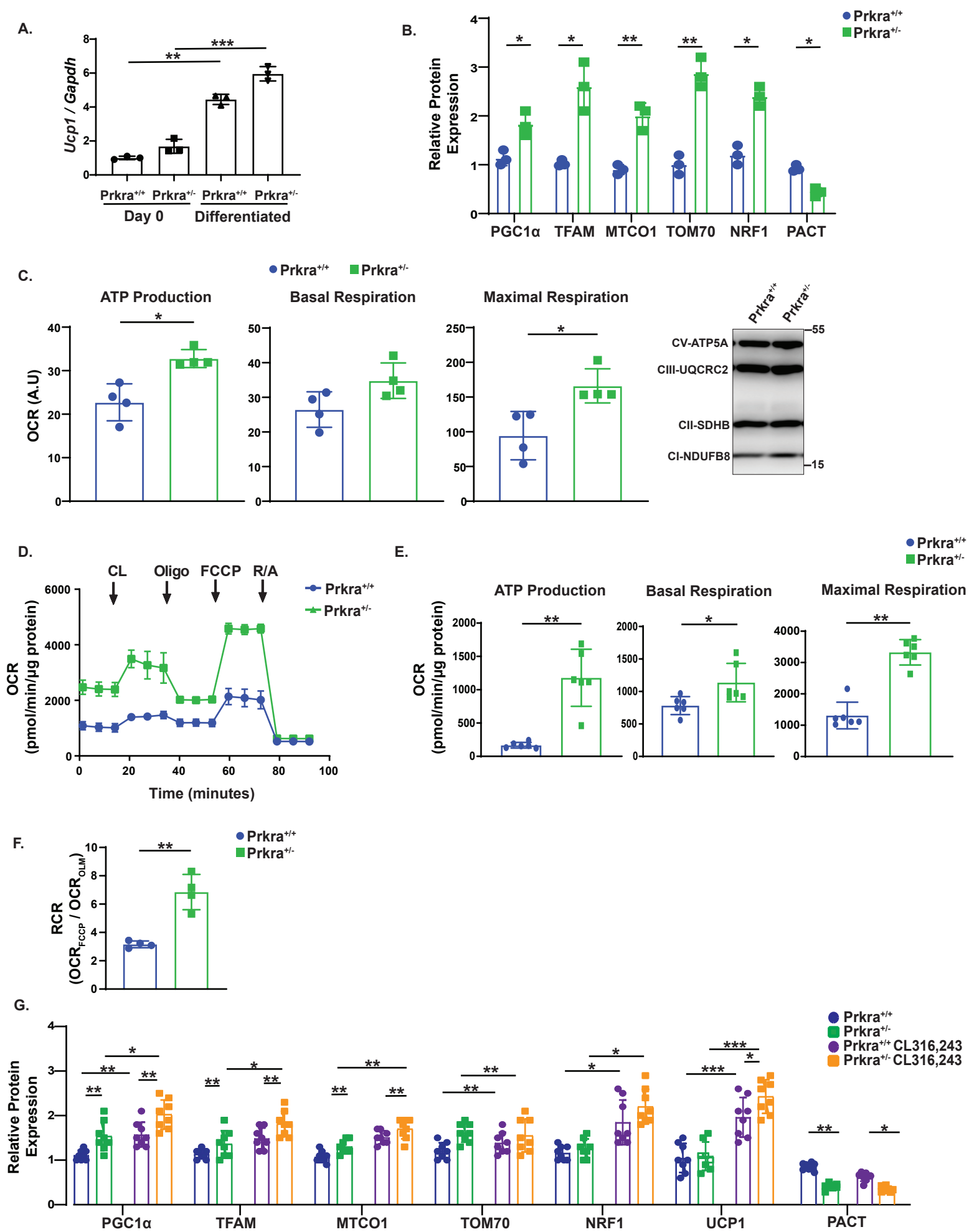

Supplement: Supplemental Figure S3A–G — PACT is required for BAT but not iWAT mitobiogenesis.A, related to Figure 4A: Primary mouse brown adipocyte total mRNA was analyzed by qRT-PCR for Ucp1 and Gapdh mRNA expression (n = 3). B, related to Figure 4A: Quantification of band intensities for PGC1α, TFAM, MTCO1, TOM70, PACT and NRF1 relative to β-actin. C, related to Figure 4C: ATP production after oligomycin (Oligo; 1 μM) injection, Maximal respiration (as the highest OCR after FCCP injection; 1 μM) and Basal Respiration (as OCR before oligomycin injection) calculated from Prkra+/+ or Prkra+/− brown adipocytes (n = 4; data were normalized to mitochondrial mass quantified from total OXPHOS protein levels (right panel) from same samples and represented as arbitrary units (A.U)). D–F, mitochondrial respiration was analyzed in Prkra+/+ or Prkra+/− primary mouse brown adipocytes by (D) oxygen consumption rate (OCR) (E) ATP production after oligomycin (Oligo; 1 μM) injection. Oligomycin: ATP synthase inhibitor; FCCP: mitochondrial uncoupler; R/A: rotenone and antimycin A mix (inhibitors for ETC complex I and III, respectively), Maximal respiration (as the highest OCR after FCCP injection; 1 μM) and Basal Respiration (as OCR before oligomycin injection). Arrows indicate time for drug injections (n = 6; data were normalized to μg of total protein). F, respiratory control ratio (RCR) was calculated from the ratio of State 3 to State 4 (OCRFCCP to OCROLIGOMYCIN) (n = 4). G, quantifications of band intensities for PGC1α, TFAM, MTCO1, TOM70, NRF1, PACT and UCP1 relative to β-actin in blots from Figure 4D (n = 8). H–K, related to mice in Figure 4D: (H) Representative H&E stained images of BAT (n = 8) (Scale bar = 50 μm). I, the water and food intake, (J) physical activity, (K) lean and fat mass of mice in Figure 4 (n = 4). L, total cell protein lysates of undifferentiated (Day 0) or differentiated iWAT adipocytes from Prkra+/+ or Prkra+/− mice were analyzed by Western blotting using specific antibodies for PGC1α, TFAM, [file mmc6.pdf]

H.

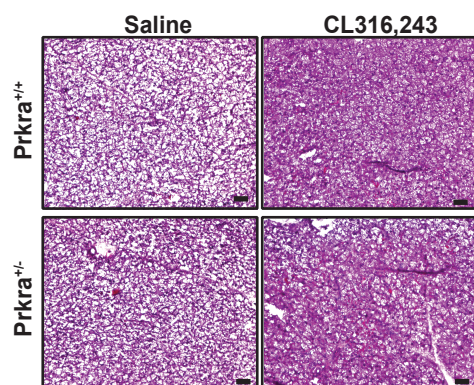

I.

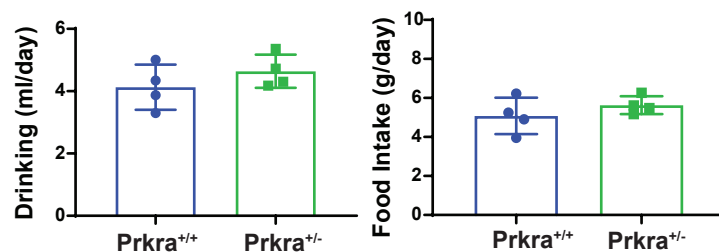

J.

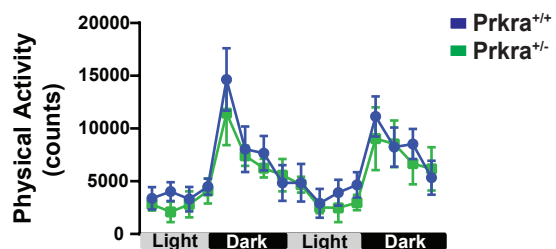

K.

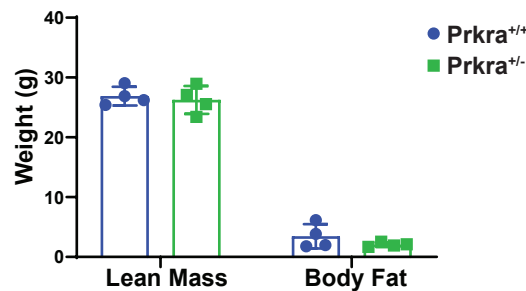

L.

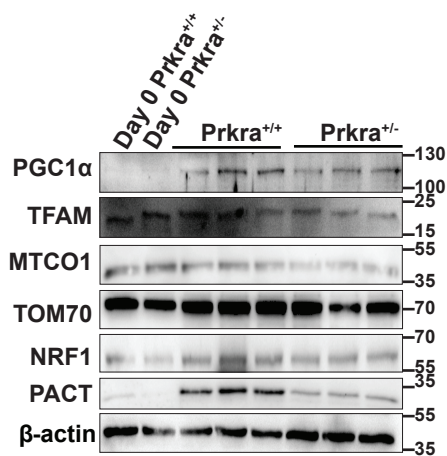

M.

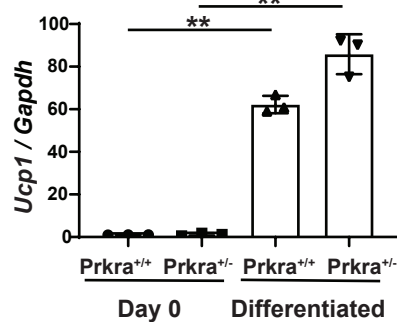

N.

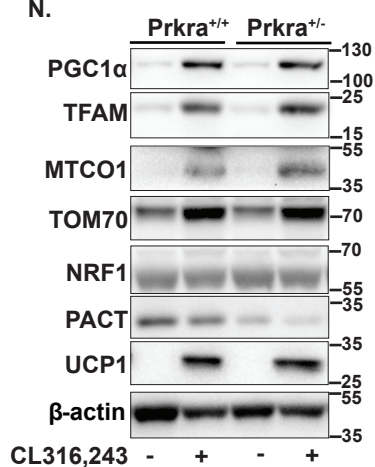

O.

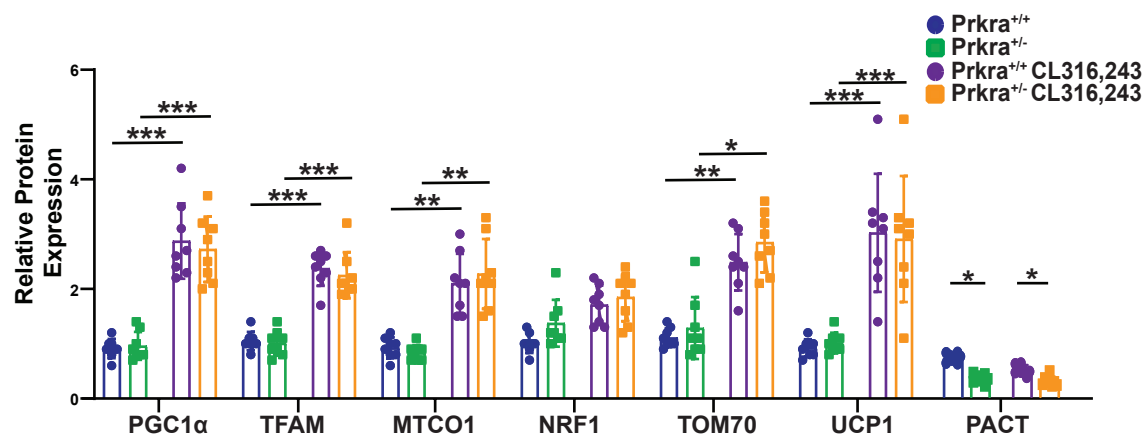

Supplement: Supplemental Figure S3H–O [file mmc7.pdf]

A.

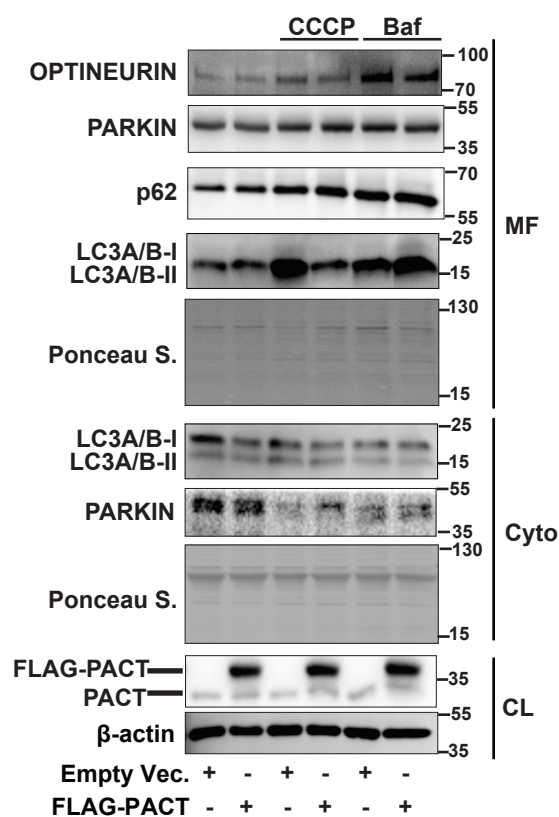

B.

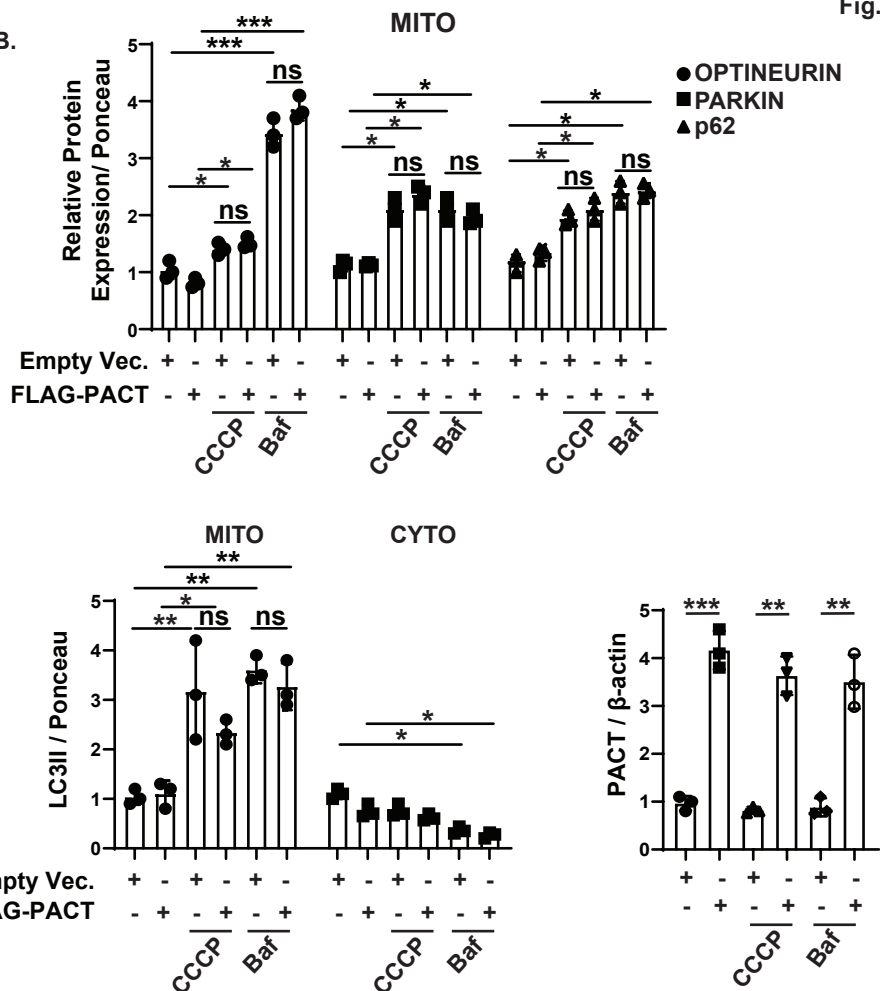

C.

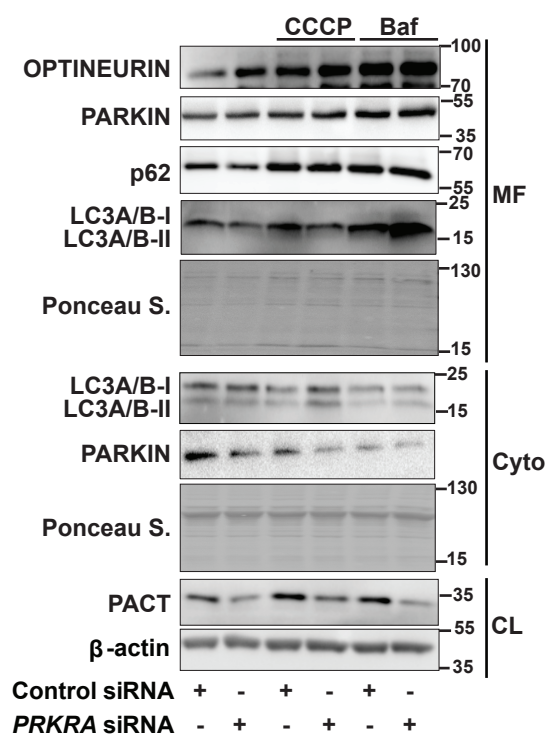

D.

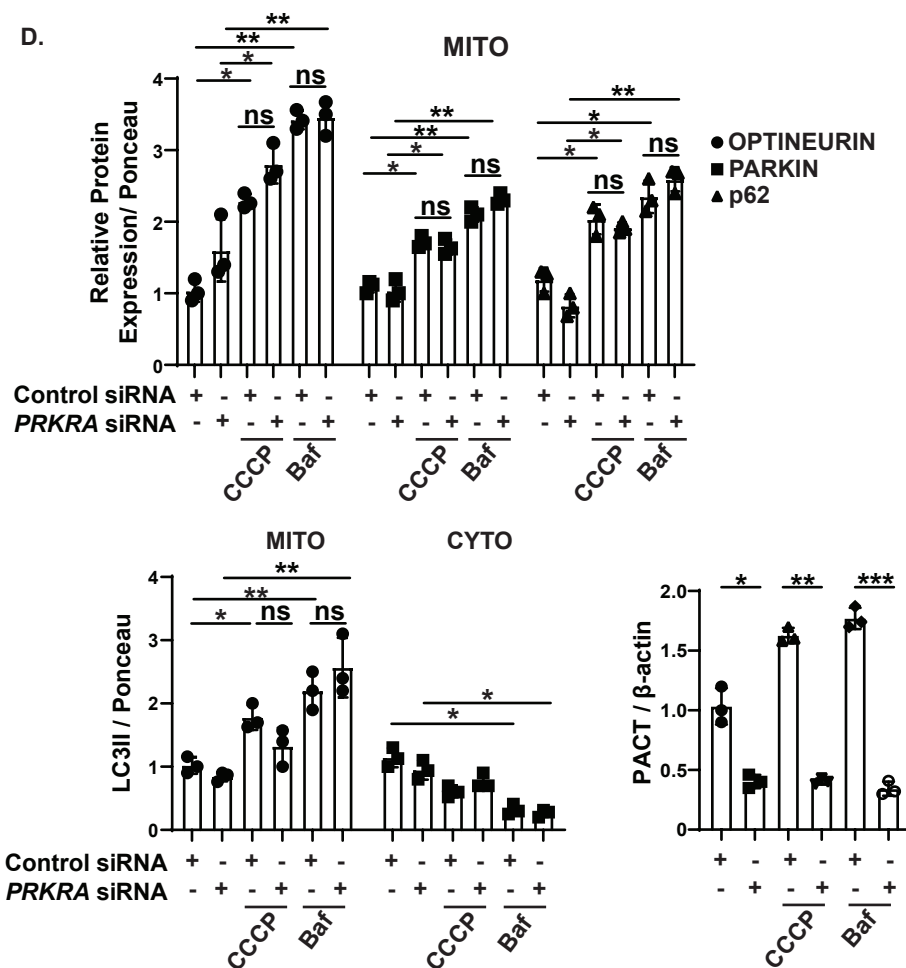

Supplement: Supplemental Figure S4A–D — PACT does not impact mitophagy or ROS scavenging enzyme protein levels.A, protein lysates from HEK293T cells that were transfected with empty vector (Empty Vec.) or FLAG-PACT plasmid and treated with CCCP (10 μM; 16 h) or Bafilomycin A1 (Baf) (50 nM; 16 h) were analyzed by Western blotting using specific antibodies for Optineurin, Parkin, p62, LC3A/B, PACT and β-actin (n = 3). B, quantification of the band intensities in (A): Optineurin, Parkin, p62 and LC3-II relative to Ponceau S. and PACT relative to β-actin (n = 3). C, protein lysates from HEK293T cells that were transfected with 100 nM Scrambled or PRKRA siRNA and treated with CCCP (10 μM; 16 h) or Bafilomycin A1 (50 nM; 16 h) were analyzed by Western blotting using specific antibodies for Optineurin, Parkin, p62, LC3A/B, PACT and β-actin (n = 3). D, quantification of band intensities in (C): Optineurin, Parkin, p62 and LC3-II relative to Ponceau S. and PACT relative to β-actin (n = 3). E–G, HEK293T cells were transfected with empty vector (Empty Vec.) or FLAG-PACT plasmid: (E) were analyzed by Western blotting using specific antibodies for CATALASE, SOD1, SOD2, PRDX3, PACT and β-actin (n = 4). F, quantifications of band intensities in (E) for CATALASE, SOD1, SOD2, PRDX3 and PACT were relative to β-actin. G, total RNA was analyzed by qRT-PCR for Glutathione synthetase (GSS), Glutathione peroxidase (GPx) and GAPDH mRNA expression (n = 5). H–J, HEK293T cells were transfected with 100 nM Scrambled or PRKRA siRNA: (H) Protein lysates were analyzed by Western blotting using specific antibodies for CATALASE, SOD1, SOD2, PRDX3, PACT and β-actin (n = 4). I, quantifications of band intensities (in G) for CATALASE, SOD1, SOD2, PRDX3 and PACT were relative to β-actin. J, total RNA was analyzed by qRT-PCR for Glutathione synthetase (GSS), Glutathione peroxidase (GPx) and GAPDH mRNA expression (n = 5). Data are mean ± SD. Unpaired t-test with Welch’s correction. ∗p ≤ 0.05, ∗∗p ≤ 0.01, ∗∗∗p ≤ 0.001, ns: not significant. CL, [file mmc8.pdf]

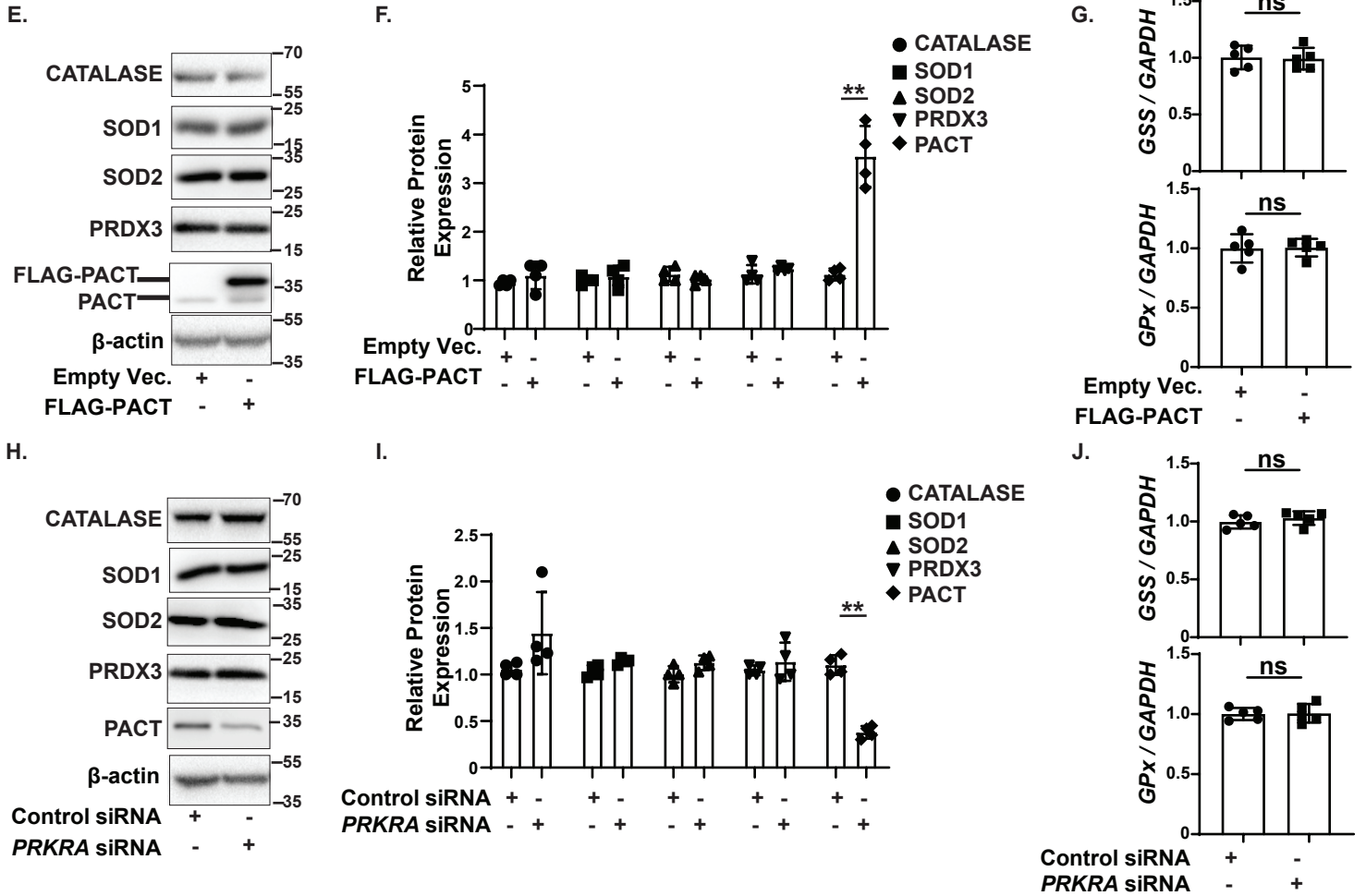

Supplement: Supplemental Figure S4E–J [file mmc9.pdf]
