# Supplementary material for: PACT establishes a posttranscriptional brake on mitochondrial biogenesis by promoting the maturation of miR-181c
Source: J Biol Chem. 2022 May 19;298(7):102050. doi: 10.1016/j.jbc.2022.102050 (PMC9218515; doi:10.1016/j.jbc.2022.102050)
Supplement: Supplemental Figure S2A–J — PACT’s control of mitobiogenesis is through miR-181c.A, total RNA from Prkra+/+ or Prkra−/− MEFs that were transfected with FLAG-PACT plasmid or Empty Vec. was analyzed by qRT-PCR for miR-181a, b, d and U6 by qRT-PCR (n = 6). B, total RNA from HEK293 cells that were transfected with scrambled or PRKRA siRNA (100 nM) was analyzed by qRT-PCR for miR-181a, b, d and U6 (n = 5). C, PRKRA and GAPDH mRNA were analyzed by qRT-PCR from total RNA in Figure 3, D and E. D, related to Figure 3G (n = 3): Protein lysates were analyzed by Western blotting using specific antibodies for TOM70 and RhoGDI. E and F, related to Figure 3, G–I: Total RNA was analyzed by qRT-PCR for (E) miR-181c and U6 RNA, (F) Quantifications of band intensities for PGC1α, TFAM, MTCO1, NRF1 and SIRT1 relative to Ponceau S (for MF) and β-actin (for CL) (G) Related to Figure 3I: Total RNA was analyzed by qRT-PCR for miR-181c and U6 (n = 3). H, related to Figure 3I: ATP production after oligomycin (Oligo; 1 μM) injection, Maximal respiration (as the highest OCR after FCCP injection; 1 μM) and Basal Respiration (as OCR before oligomycin injection) calculated from MEFs transfected with scrambled or miR-181c-5p mimic (100 nM) (n = 5; data were normalized to mitochondrial mass quantified from total OXPHOS protein levels (right panel) from same samples and represented as arbitrary units (A.U)). I and J, HEK293T cells were transfected with scrambled or miR-181c mimic (100 nM); (I) OCR was measured (n = 5; data were normalized to μg of total protein). J, ATP production after oligomycin (Oligo; 1 μM) injection, Maximal respiration (as the highest OCR after FCCP injection; 1 μM) and Basal Respiration (as OCR before oligomycin injection) were calculated. K, respiratory control ratio (RCR) of HEK293T cells transfected with scrambled or miR-181c mimic (100 nM) was calculated from the ratio of State 3 to State 4 (OCRFCCP to OCROLIGOMYCIN) (n = 5). L, related to Figure 3J: Protein lysates were analyzed by Western blotting [file mmc3.pdf]

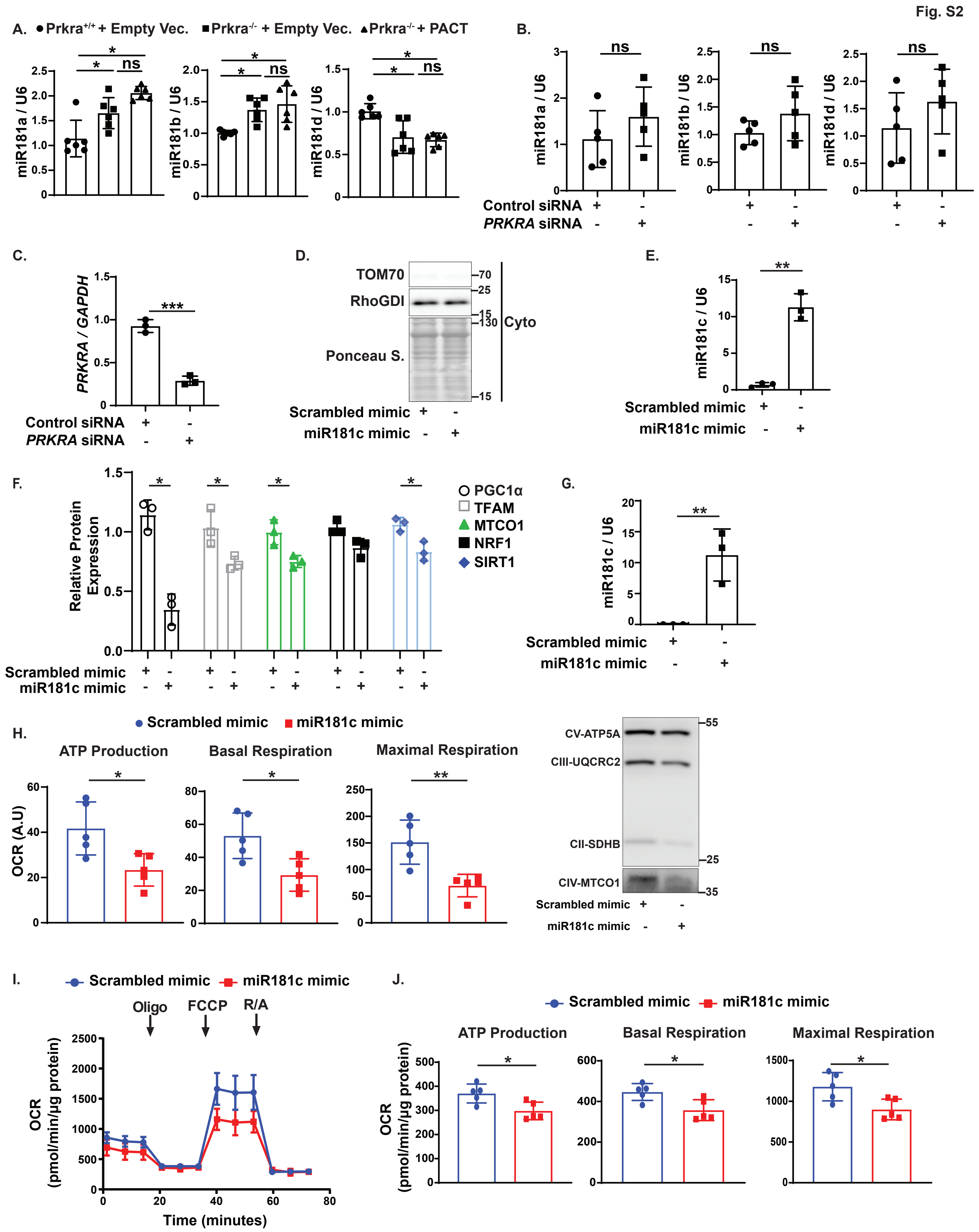
Fig. S2
A. Prkra+/+ + Empty Vec. Prkra-/- + Empty Vec. Prkra-/- + PACT
miR181a / U6
miR181b / U6
miR181d / U6
ns
B.
Control siRNA
PRKRA siRNA
C.
PRKRA / GAPDH
D.
TOM70
RhoGDI
Ponceau S.
Cyto
Scrambled mimic
miR181c mimic
E.
miR181c / U6
F.
Relative Protein Expression
PGC1α
TFAM
MTCO1
NRF1
SIRT1
G.
H.
ATP Production
Basal Respiration
Maximal Respiration
OCR (A.U)
CV-ATP5A
CIII-UQCRC2
CII-SDHB
CIV-MTCO1
I.
Oligo
FCCP
R/A
OCR (pmol/min/μg protein)
Time (minutes)
J.
